# Integrated Biological and Chemical Investigation of Indonesian Marine Organisms Targeting Anti-Quorum-Sensing, Anti-Biofilm, Anti-Biofouling, and Anti-Biocorrosion Activities

**DOI:** 10.3390/molecules30061202

**Published:** 2025-03-07

**Authors:** Novriyandi Hanif, Jihan Azmi Miftah, Henny Dwi Yanti, Emmanuel Tope Oluwabusola, Vira Amanda Zahra, Nurul Farhana Salleh, Binu Kundukad, Lik Tong Tan, Nicole J. de Voogd, Nisa Rachmania, Marcel Jaspars, Staffan Kjelleberg, Dedi Noviendri, Anggia Murni, Junichi Tanaka

**Affiliations:** 1 Department of Chemistry, Faculty of Mathematics and Natural Sciences, IPB University, Bogor 16680, Indonesia; jihanazmi@apps.ipb.ac.id (J.A.M.); viraamandazahra@gmail.com (V.A.Z.); 2 Tropical Biopharmaca Research Center, IPB University, Bogor 16128, Indonesia; dyantihenny@gmail.com (H.D.Y.); anggia_murni@apps.ipb.ac.id (A.M.); 3 Marine Biodiscovery Centre, Department of Chemistry, University of Aberdeen, Aberdeen AB24 3FX, UK; emmanuel.oluwabusola3@abdn.ac.uk (E.T.O.); m.jaspars@abdn.ac.uk (M.J.); 4 Natural Sciences and Science Education, National Institute of Education, Nanyang Technological University, 1 Nanyang Walk, Singapore 637616, Singapore; nie21.nfs@e.ntu.edu.sg (N.F.S.); liktong.tan@nie.edu.sg (L.T.T.); 5 Singapore Center for Environmental Life Sciences Engineering (SCELSE), Nanyang Technological University, 60 Nanyang Drive, SBS-01N-27, Singapore 637551, Singapore; binu.kundukad@ntu.edu.sg (B.K.); laskjelleberg@ntu.edu.sg (S.K.); 6 Naturalis Biodiversity Center, P.O. Box 9517, 2300 RA Leiden, The Netherlands; nicole.devoogd@naturalis.nl; 7 Institute of Biology (IBL), Leiden University, P.O. Box 9505, 2300 RA Leiden, The Netherlands; 8 Department of Biology, Faculty of Mathematics and Natural Sciences, IPB University, Bogor 16680, Indonesia; nrachmania@apps.ipb.ac.id; 9 School of Biological Sciences, Nanyang Technological University, 60 Nanyang Drive, Singapore 637551, Singapore; 10 School of Biological, Earth and Environmental Sciences, University of New South Wales, Sydney, NSW 2033, Australia; 11 Research Center for Pharmaceutical Ingredients and Traditional Medicine, BRIN Cibinong-Bogor, Bogor 16911, Indonesia; dedi025@brin.go.id; 12 Department of Chemistry, Biology and Marine Science, University of the Ryukyus, Okinawa 903-0213, Japan; jtanaka@sci.u-ryukyu.ac.jp

**Keywords:** anti-quorum sensing, anti-biofilm, anti-biofouling, anti-biocorrosion, molecular networking, NMR, *Sigilina* cf. *signifera*, *Lamellodysidea herbacea*

## Abstract

Microorganisms play a significant role in biofouling and biocorrosion within the maritime industry. Addressing these challenges requires an innovative and integrated approach utilizing marine natural products with beneficial properties. A comprehensive screening of 173 non-toxic EtOAc and H₂O extracts derived from diverse marine organisms collected in Indonesian waters was conducted using a robust panel of assays. These included antimicrobial tests and classical biosurfactant assays (drop collapse and oil displacement), as well as anti-quorum-sensing (QS) and anti-biofilm assays. These screening efforts identified five active extracts with promising activities. Among these, EtOAc extracts of the marine tunicate *Sigilina* cf. *signifera* (0159-22e) and the marine sponge *Lamellodysidea herbacea* (0194-24c) demonstrated significant anti-biofouling activity against *Perna indica* and anti-biocorrosion performance (mpy 10.70 ± 0.70 for *S.* cf. *signifera*; 7.87 ± 0.86 for *L. herbacea;* 13.60 ± 1.70 for positive control Tetracorr CI-2915). Further chemical analyses of the active extracts, including LC-HR-MS/MS, MS-based molecular networking, and chemoinformatics, revealed the presence of both known and new bioactive compounds. These included tambjamines and polybrominated diphenyl ethers (PBDEs), which are likely contributors to the observed bioactivities. Subsequent investigations uncovered new anti-QS and anti-biofilm properties in synthetic and natural PBDEs **1**–**12** previously derived from *L. herbacea*. Among these, **8** exhibited the most potent anti-QS activity, with an IC_50_ value of 15 µM, while **4** significantly reduced biofilm formation at a concentration of 1 µM. This study highlights the potential of marine-derived compounds in addressing biofouling and biocorrosion challenges in a sustainable and effective manner.

## 1. Introduction

Biofouling and biocorrosion of underwater structures are closely linked issues that significantly affect the maritime industry. These phenomena result in substantial annual economic losses across key activities, such as shipping, aquaculture, desalination, marine renewable energy, and the global spread of invasive marine species [1]. Jin et al. (2022) estimated that biofouling alone costs the maritime industry over USD 15 billion annually, while the global cost of corrosion reaches USD 2.5 trillion per year. Notably, approximately 20% of corrosion in aquatic systems is attributed to biofouling, specifically a process known as microbially influenced corrosion (MIC) [2]. Moreover, the settlement of fouling organisms on surfaces alters the chemical and physical properties of their surroundings, leading to material and structural biodegradation. These changes include variations in ion composition and concentration, dissolved oxygen levels, pH, liquid conductivity, and the promotion of chemical and electrochemical reactions.

Marine biofouling [3,4] begins with the attachment of microorganisms, such as bacteria, to submerged surfaces, where they aggregate to form microbial films. Quorum sensing (QS), a specialized communication mechanism among bacteria, provides biofilms with mechanical stability and facilitates their maturation. Once formed, these biofilms attract the settlement of algal spores and the larvae of marine invertebrates, such as barnacles, mussels, and hard corals, which subsequently grow in abundance. Biofouling accelerates corrosion and contributes to surface degradation and structural failure.

Biofouling and biocorrosion are interconnected challenges that cannot be effectively addressed with traditional single-function antifouling or anticorrosion materials. Addressing these issues requires an integrated anti-biofouling and anti-biocorrosion strategy, leveraging marine natural products. Key considerations for such an approach include 1. environmentally friendly and biodegradable molecules with minimal toxicity; 2. disruption of bacterial communication via the quorum-sensing (QS) mechanism to minimize resistance; 3. biosurfactant properties to inhibit microbial adhesion; and 4. sequential activity targeting biofilm formation, biofouling, and biocorrosion. This comprehensive strategy is essential for sustainable and effective solutions.

Traditional synthetic antifoulants, including tributyltin oxide (TBT), Sea-Nine™ 211, Irgarol^®^ 1051, and Diuron, have been deemed unsuitable due to their environmental toxicity [3]. Recently, newer synthetic biocide-based antifoulants, such as Econea^®^ and Selektope^®^, have gained approval [1]. In contrast, marine organisms have naturally evolved chemical defenses to protect themselves from fouling by other organisms [5]. Many of these organisms produce complex antifouling secondary metabolites, which offer a direct source of inspiration for scalable antifouling solutions [2,6,7,8]. Observations of sessile marine organisms, whose surfaces often remain free of fouling, help identify those that biosynthesize effective antifouling compounds. To date, over 500 marine natural products with antifouling activity have been isolated worldwide [6,7,8,9]. Several compounds have demonstrated QS-inhibiting antifouling properties, including brominated furanones [6], aplyzanzine E, bromine-containing 2-aminoimidazole compounds [10], and cembrane-type molecules [7]. These discoveries underscore the potential of marine natural products in developing sustainable antifouling technologies.

Currently, no integrated program exists to address both anti-biofouling and anti-biocorrosion using marine natural products. This is despite their potential for combining antifouling and anticorrosion approaches as promising strategies [1,2]. Biocide-based coatings, while effective in preventing marine biofouling, have been shown to negatively impact marine ecosystems [11]. This study proposes a novel integrated strategy for identifying marine natural products derived from Indonesian marine organisms with both anti-biofouling and anti-biocorrosion properties, aiming to address critical challenges in the maritime industry and environmental conservation. To achieve this, a comprehensive and systematic experimental design was developed, including the incorporation of assays such as a brine shrimp lethality assay against *Artemia salina* larvae, an antibacterial assay against *Staphylococcus aureus* and *Pseudomonas aeruginosa*, a biosurfactant assay using drop-collapse and oil displacement methods, an anti-QS assay based on the *P. aeruginosa* PAO1 *lasB-gfp* reporter strain, an anti-biofilm assay with *P. aeruginosa*, an anti-biofouling assay using *P. indica*, and an anti-biocorrosion assay with a modified corrosion coupon. Additionally, the molecular characteristics of active marine-derived extracts originating from the marine tunicate *Sigilina* cf. *signifera*, the marine sponges *Lamellodysidea herbacea*, *Haliclona* sp., and *Agelas* sp., and a marine cyanobacterium cf. *Lyngbya* sp. were analyzed using techniques such as LC-HR-MS/MS, HR-MS-based molecular networking (MN), and chemoinformatics. Furthermore, a series of synthetic and natural antibacterial polybrominated diphenyl ethers (PBDEs), **1**–**12**, derived from *L. herbacea*, were evaluated for their anti-QS and anti-biofilm activities. A preliminary structure–activity relationship (SAR) for anti-QS PBDEs was also proposed. The integrated approach adopted in this study highlights the potential of marine-derived compounds in developing sustainable solutions to address biofouling and biocorrosion.

## 2. Results and Discussion

### 2.1. Toxicity of Marine-Derived Extracts Based on the Brine Shrimp (Artemia salina) Lethality Assay

Some antifoulants have been deemed environmentally toxic based on the *A. salina* lethality assay and are banned from commercial use. These include tributyltin oxide (TBTO, LC_50_ = 8.7 µg/mL), copper (II) sulfate (CuSO_4_, LC_50_ = 6.2 µg/mL) [12], tributyl tin (TBT, LC_50_ = 0.208 × 10⁻^3^ µg/mL), Diuron (LC_50_ = 10.261 µg/mL), and Irgarol^®^ 1051 (LC_50_ = 0.982 µg/mL) [13]. As such, screening marine-derived extracts for brine shrimp lethality was a critical initial step in identifying non-toxic candidates in this study.

**Figure 1:**
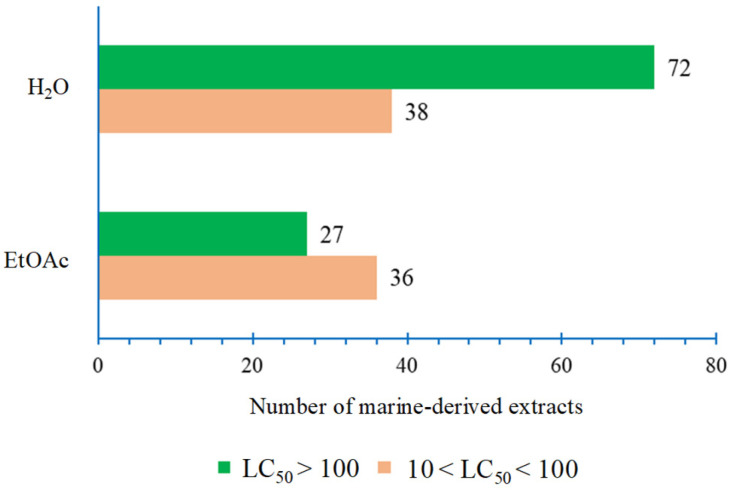
Toxicity distribution of Indonesian marine-derived extracts into two categories of toxicity against brine shrimp larvae: 10 µg/mL < LC_50_ < 100 µg/mL and LC_50_ > 100 µg/mL.

A brine shrimp (*A. salina*) lethality assay was used to screen EtOAc and H_2_O extracts from 277 Indonesian marine organisms, identifying 173 extracts meeting two key non-toxicity criteria: (1) LC_50_ values between 10 µg/mL and 100 µg/mL and (2) LC_50_ values greater than 100 µg/mL (Figure 1). These criteria were essential for discovering non-toxic molecules with anti-biofouling and anti-biocorrosion properties in subsequent screening efforts in this study. Among these, 36 EtOAc extracts fulfilled the first criterion (10 µg/mL < LC_50_ < 100 µg/mL), while 27 were grouped under the second criterion (LC_50_ > 100 µg/mL). Similarly, 38 H_2_O extracts met the first criterion, and 72 the second.

**Figure 2:**
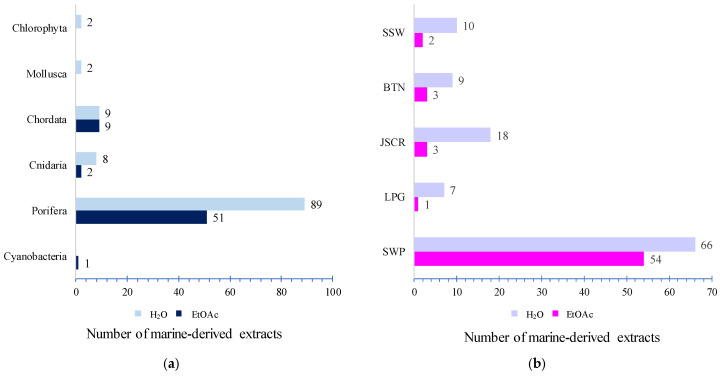
Distribution of Indonesian marine-derived extracts based on biological sources (**a**) and their biogeographic distribution (**b**). SWP = Southwest Papua; LPG = Lampung; JSCR = Jakarta Special Capital Region; BTN = Banten; SSW = South Sulawesi.

The marine organisms producing low-toxicity extracts included sponges, tunicates, soft corals, algae, nudibranchs, and cyanobacteria. Sponges were the most significant source, followed by tunicates and soft corals (Figure 2a). Most of these organisms were collected from Southwest Papua (SWP), located at the heart of the Coral Triangle, which supports an abundance of sponges and tunicates (Figure 2b).

### 2.2. Antibacterial Activity of Marine-Derived Extracts Against S. aureus and P. aeruginosa

Screening 173 EtOAc and H_2_O extracts with LC_50_ > 10 µg/mL against Gram-positive *S. aureus* and Gram-negative *P. aeruginosa* yielded 14 hits: 4 from EtOAc extracts and 10 from H_2_O extracts (Table 1). Additionally, 22 extracts showed activity exclusively against *S. aureus* (6 from EtOAc and 16 from H_2_O extracts), while 144 extracts were active only against *P. aeruginosa* (44 from EtOAc and 100 from H_2_O extracts). A total of 29 extracts were inactive against both *S. aureus* and *P. aeruginosa*.

The 14 extracts exhibiting antibacterial activity against both *S. aureus* and *P. aeruginosa*, along with 1 EtOAc extract from *L. herbacea* active against *S. aureus*, were selected for further evaluation using classical biosurfactant assays. The marine sponge *L. herbacea* was prioritized for in-depth investigation due to its bioactive polybrominated diphenyl ethers (PBDEs), which show promise as anti-quorum-sensing (anti-QS), anti-biofilm, anti-biofouling, and anti-biocorrosion agents. Previous studies have demonstrated the antifouling activity of PBDEs from *L. herbacea* against marine bacteria, diatoms, barnacle larvae, and juvenile mussels [14].

In summary, the 15 extracts included 12 from sponges [*Haliclona* sp. (3 samples), *Plakortis* sp. (1 sample), *Agelas* sp. (2 sample), *Niphates* sp. (1 sample), *Acanthostrongylophora ingens* (2 samples), *Neopetrosia* sp. (1 sample), *Clathria* sp. (1 sample), and *L. herbacea* (1 sample)], 1 from cf. *Lyngbya* sp., and 2 from tunicates [*Sigilina* cf. *signifera* and an unidentified tunicate (0126-22e)]. These extracts were confirmed to be non-toxic to *A. salina* larvae, with LC_50_ > 10 µg/mL (Table 1).

### 2.3. Biosurfactant Properties of Marine-Derived Extracts

Fifteen marine extracts, previously selected due to their significant antibacterial activity (Table 1), were screened for biosurfactant properties using two classical methods: drop-collapse and oil displacement assays. Nine of the extracts demonstrated enhanced drop-collapse activity on mineral and olive oil surfaces compared to the positive control (Figure S1). Similarly, in the oil displacement assay, nine extracts exhibited superior biosurfactant activity with mineral oil, while eight showed better activity with olive oil (Figure S1). The experiments included a positive control (1.42% SDS) and a negative control (60% aqueous MeOH).

Overall, nine marine-derived extracts (Table 2) were identified as potential sources of biosurfactant molecules. These included four EtOAc extracts from *Haliclona* sp. (0178-22e, SWP), cf. *Lyngbya* sp. (0076-18c, BTN), *S.* cf. *signifera* (0159-22e, SWP), and *L. herbacea* (0194-24c, BTN). Additionally, five H_2_O extracts were identified from *Agelas* sp. (0036-22e, SWP), *Niphates* sp. (0002-22e, SWP), *Neopetrosia* sp. (0021-22e, SWP), *Agelas* sp. (0049-16b, JSCR), and *Haliclona* sp. (0015-22e, SWP). Biosurfactants are known to inhibit biofilm formation due to their antimicrobial activity [4]. Consequently, the extracts containing biosurfactant properties are expected to exhibit anti-quorum-sensing (anti-QS), anti-biofilm, and anti-biofouling properties, with potential applications as anti-biocorrosion agents.

### 2.4. Anti-QS Activity of Selected Marine-Derived Extracts

The fifteen previously selected marine-derived extracts (Table 1) were screened for anti-QS activity using the *P. aeruginosa* PAO1 *lasB*-*gfp* bioreporter strain at a single dose of 100 µg/mL (Table S1). In the bacterial reporter strain, the production of the green fluorescence protein (GFP) is indicative of QS induction [15]. The QS-inhibitory activity of the extracts or compounds is assessed by measuring the reduction in GFP production compared to the bacterial control. GFP expression was measured in relative fluorescence units and normalized by dividing the GFP values by the corresponding OD_600_ values measured at different time points.

**Figure 3:**
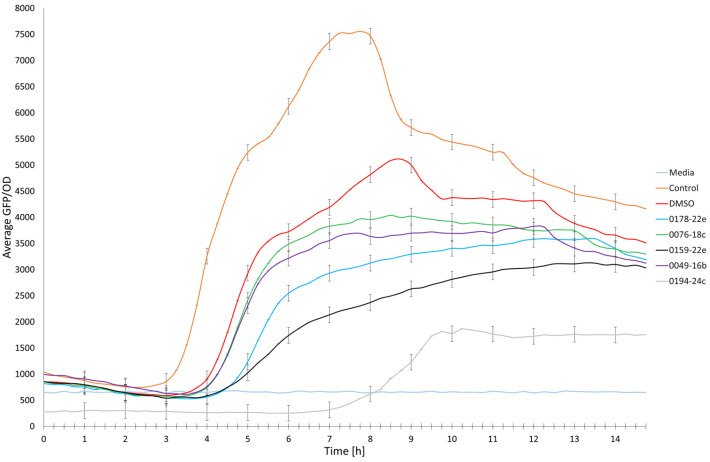
Single-dose inhibition curves (100 µg/mL) of five marine-derived extracts, including EtOAc extracts of *Haliclona* sp. (0178-22e), cf. *Lyngbya* sp. (0076-18c), *S.* cf. *signifera* (0159-22e), and *L. herbacea* (0194-24c) and the H_2_O extract of *Agelas* sp. (0049-16b), incubated with the QS bioreporter strain PAO1 *lasB-gfp*. The experiments were conducted in triplicate.

Among the fifteen extracts, only five extracts, including EtOAc extracts of *Haliclona* sp. (0178-22e), cf. *Lyngbya* sp. (0076-18c), *S.* cf. *signifera* (0159-22e), and *L. herbacea* (0194-24c) and the H_2_O extract of *Agelas* sp. (0049-16b), showed possible QS-inhibitory activity (Figure 3). The most active marine-derived extract tested at 100 µg/mL was from *L. herbacea* (0194-24c), which showed 50% inhibition. This was followed by *S.* cf. *signifera* (0159-22e) (45% inhibition), *Haliclona* sp. (0178-22e) (39% inhibition), *Agelas* sp. (0049-16b) (33% inhibition), and cf. *Lyngbya* sp. (0076-18c) (29% inhibition). Even though the H_2_O extract of *Niphates* sp. (0002-22e) showed a decrease in fluorescence expression, its inhibition was due to antibiotic activity and not QS inhibition. As shown in Table 2, the five extracts with anti-QS activity also demonstrated biosurfactant properties. Based on the anti-QS assay, these five anti-QS marine-derived extracts are hypothesized to have potential anti-biofilm, anti-biofouling, and anti-biocorrosion activities.

Previous studies have shown that the extracts of sponge species related to the *Haliclona* sp. (0178-22e) and *Agelas* sp. (0049-16b) used in this study possess quorum-sensing-inhibitory activities. For instance, the methanolic extract of *Haliclona* (*Gellius*) *megastoma* (TS 25) collected from Palk Bay, India, inhibited acyl homoserine lactone-mediated violacein production in *Choromobacterium violaceum* (ATCC 12472) and CV026 [16]. Several alkaloids, such as ageliferin, mauritamide B, midpacamide, and 4-(4,5-dibromo-1-methyl-1H-pyrrole-2-carboxamido) butanoic acid isolated from *Agelas conifer*, *A. nakamurai*, *A. mauritiana*, and *Agelas* sp., respectively, inhibited QS of the bacterial reporter *C. violaceum* CV017, with MIC values ranging from 11.29 to 458.61 µM [17]. In addition, marine cyanobacteria, including *Lyngbya* species, are known to produce anti-quorum-sensing inhibitors, such as lyngbyoic acids and malyngolide [18]. Despite other biological activities reported for extracts of *S. signifera* [19] and *L. herbacea* [20], their quorum-sensing-inhibitory activities have never been reported.

### 2.5. Anti-Biofilm Activity of Marine-Derived Extracts

**Figure 4:**
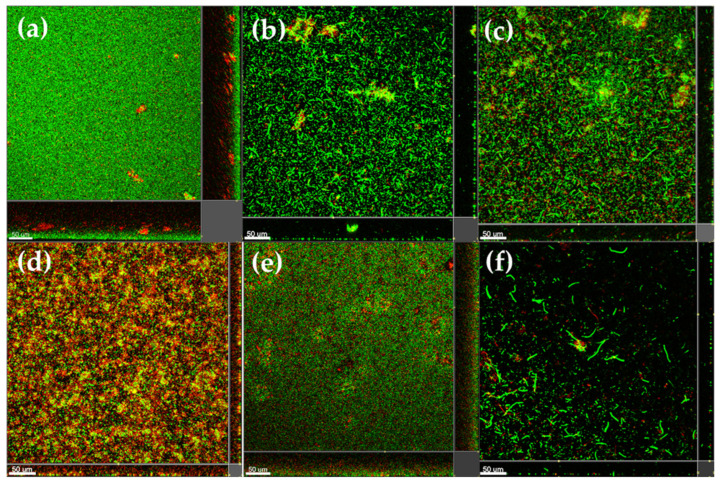
The anti-biofilm activity of the five marine-derived extracts based on *P. aeruginosa* imaged by confocal microscope. The negative control (**a**), EtOAc extracts of *Haliclona* sp. (0178-22e) (**b**), cf. *Lyngbya* sp. (0076-18c) (**c**), *S.* cf. *signifera* (0159-22e) (**d**), and *L. herbacea* (0194-24c) (**e**), and the H_2_O extract of *Agelas* sp. (0049-16b) (**f**). The concentration used for each marine-derived extract was 1000 µg/mL.

**Figure 5:**
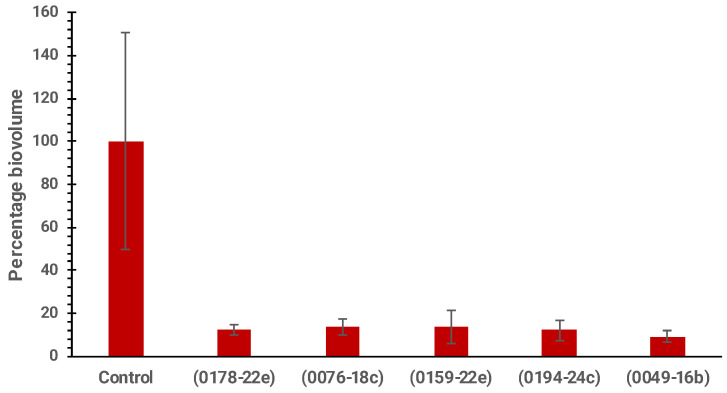
Biovolume percentages of *P. aeruginosa* biofilms incubated with five marine-derived extracts, including EtOAc extracts of *Haliclona* sp. (0178-22e), cf. *Lyngbya* sp. (0076-18c), *S.* cf. *signifera* (0159-22e), and *L. herbacea* (0194-24c) and the H_2_O extract of *Agelas* sp. (0049-16b). Each extract was tested at 1000 µg/mL.

Next, we investigated the anti-biofilm activity of the five anti-QS marine extracts, including EtOAc extracts of *Haliclona* sp. (0178-22e), cf. *Lyngbya* sp. (0076-18c), *S.* cf. *signifera* (0159-22e), and *L. herbacea* (0194-24c) and the H_2_O extract of *Agelas* sp. (0049-16b). The anti-biofilm activity was evaluated by cultivating *P. aeruginosa* biofilms in the presence of 1000 µg/mL of each extract. Confocal microscopy images revealed a notable absence of biofilm formation (Figure 4). Further, quantitative analysis of the biovolume from these images demonstrated a significant reduction in biofilm biovolume by EtOAc extracts of *Haliclona* sp. (87.7%), cf. *Lyngbya* sp. (86.4%), *S.* cf. *signifera* (86.1%), and *L. herbacea* (88.0%) and the H_2_O extract of *Agelas* sp. (90.8%) (Figure 5), highlighting the potent anti-biofilm effects of these five marine-derived extracts.

The extracts of several sponge species related to *Haliclona* sp. (0178-22e) and *Agelas* sp. (0049-16b) have been reported to have anti-biofilm activity. For instance, extracts of *Haliclona caerulea*, collected from Pichilingue, Mexico, prevented the adhesion of several biofilm-forming bacteria, including *Vibrio* sp., *Pseudoalteromonas* sp., *Bacillus subtilis*, and *B. pumilus* [21]. In addition, the methanolic extract of the sponge *Agelas dispar* was shown to inhibit biofilm formation by *Candida krusei* (ATCC 6258), *C. glabrata* (ATCC 2001), and *C. parapsilosis* [22]. Alkaloids such as bromoageliferin (from *A. dilatata*) and an oxime derivative of agelasine D (from *A. nakamurai*) have been reported to have anti-biofilm activity against *P. aeruginosa* and *Staphylococcus epidermidis*, respectively [17,23]. Interestingly, from molecular networking analysis, a cluster related to the agelasine molecular family was detected, and it could be responsible for the anti-biofilm activity observed in the extracts of *Agelas* sp. (0049-16b). Moreover, extracts from marine cyanobacteria, including *Lyngbya* sp., have been reported to possess anti-biofilm activity against several microbial strains [24,25]. Similar to the quorum-sensing-inhibitory assay above, the anti-biofilm activities of *S. signifera* and *L. herbacea* have not been reported.

### 2.6. Anti-Biofouling Activity of Marine-Derived Extracts

**Figure 6:**
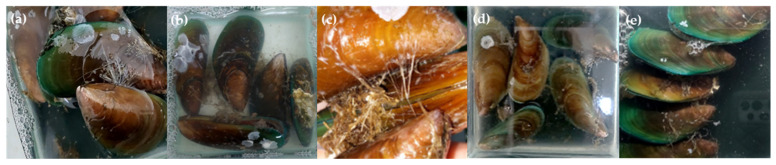
Anti-biofouling activity of marine-derived extracts using *P. indica*. Negative control (**a**), positive control CuSO_4_ (**b**), EtOAc extract of *S.* cf. *signifera* (0159-22e) at 25 µg/mL (**c**), EtOAc extract of *S.* cf. *signifera* (0159-22e) at 100 µg/mL (**d**), EtOAc extract of *L. herbacea* (0194-24c) at 100 µg/mL (**e**).

Due to limited material, the anti-biofouling activities of five marine-derived extracts from *Haliclona* sp. (0178-22e), cf. *Lyngbya* sp. (0076-18c), *S.* cf. *signifera* (0159-22e), *L. herbacea* (0194-24c), and *Agelas* sp. (0049-16b) were qualitatively evaluated using the mussel *Perna indica* at concentrations of 25 and 100 µg/mL. As shown in Figure 6, the EtOAc extracts of *S.* cf. *signifera* (0159-22e) and *L. herbacea* showed significant anti-biofouling activity only at 100 µg/mL, while the remaining three extracts exhibited weak activity at 25 and 100 µg/mL. Additionally, the extracts of *S.* cf. *signifera* (0159-22e) and *L. herbacea* (0194-24c) effectively prevented *P. indica* byssus adherence without causing significant lethality. In contrast, the commercial positive control (CuSO_4_) demonstrated anti-biofouling activity but was lethal at a concentration of 25 µg/mL. It is proposed that the effective concentration for the anti-biofouling activity of EtOAc extracts of *S.* cf. *signifera* (0159-22e) and *L. herbacea* (0194-24c) is between 25 and 100 µg/mL.

The results from the anti-biofouling assay of the five marine-derived extracts in this study showed their potential use in controlling macrobiofoulers, such as mussels. For example, the formulation of extracts from *Haliclona caerulea* and the macroalga *Sargassum horridum* revealed a significant reduction in biofouling in field testing [21]. Chemical analysis has also led to the identification of antifouling molecules from *Haliclona* and *Agelas* species, such as haliclonacyclamine A, halaminol A, bis-1-oxaquinolizidine, and agelasine alkaloids [17,26,27]. Agelasine alkaloids have also been detected in extracts of *Agelas* sp. (0049-16b) and could be responsible for the anti-biofouling activity in this study.

### 2.7. Anti-Biocorrosion of Marine-Derived Extracts

To screen the five marine-derived extracts for biocorrosion inhibitory activity, a modified coupon corrosion analysis was performed at two concentrations, 35 and 70 µg/mL (Table 3). Three marine-derived extracts, including EtOAc extracts of *S.* cf. *signifera* (0159-22e) and *L. herbacea* (0194-24c), as well as the H_2_O extract of *Agelas* sp., exhibited significant anti-biocorrosion activity when tested at 35 µg/mL. At the higher concentration of 70 µg/mL, all extracts showed significant anti-biocorrosion activity. In particular, extracts of *S.* cf. *signifera* (0159-22e) and *L. herbacea* (0194-24c) exhibited corrosion rates (mpy, 35 µg/mL) of 10.70 ± 1.70 and 7.87 ± 0.86, while at 70 µg/mL, their corrosion rates (mpy) were 7.72 ± 1.74 and 4.64 ± 0.52, respectively. In comparison, the positive control Tetracorr CI-2915 showed corrosion rates (mpy) of 13.60 ± 1.70 (35 µg/mL) and 12.00 ± 1.70 (70 µg/mL). The systematic investigation identified five marine-derived extracts with significant anti-biofouling and anti-biocorrosion activities. Among these, two extracts, derived from *S.* cf. *signifera* and *L. herbacea*, exhibited promising green-corrosion-inhibitory properties at low concentrations. These findings revealed new sources of green marine corrosion inhibitors in addition to plants, marine algae, and microbes [28,29,30].

### 2.8. LC-HR-MS/MS, HRMS-Based Molecular Networking, and Chemoinformatic Database Analyses

**Figure 7:**
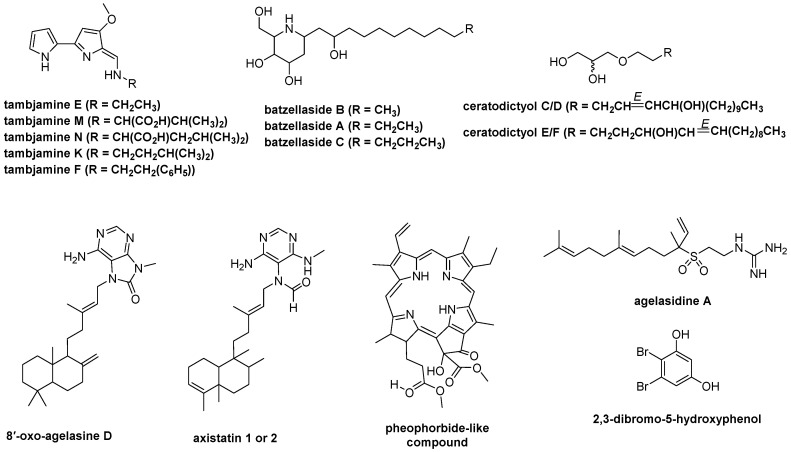

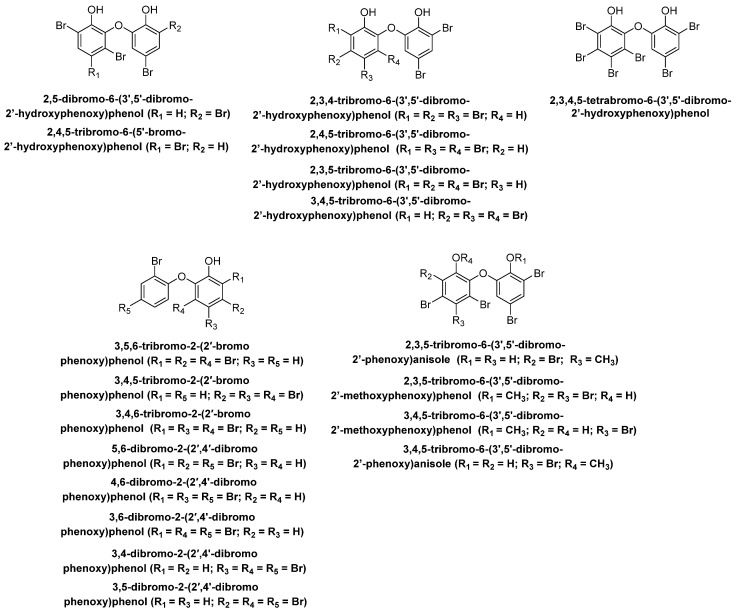
Possible known molecules observed by LC-HR-MS/MS analysis of the five marine-derived extracts, including EtOAc extracts of *S.* cf. *signifera* (0159-22e), *L. herbacea* (0194-24c), *Haliclona* sp. (0178-22e), and cf. *Lyngbya* sp. (0076-18c) and the H_2_O extract of *Agelas* sp. (0049-16b).

To uncover the compounds potentially responsible for the observed biological activities, an integrated approach involving LC-HR-MS/MS, HRMS-based molecular networking, and chemoinformatic analyses of the five bioactive marine-derived extracts was performed. LC-HR-MS/MS with TOF-MS (positive mode) and Orbitrap MS (negative mode), as well as chemoinformatic analyses using the Marinlit database on extracts of *S.* cf. *signifera* (0159-22e), *L. herbacea* (0194-24c), *Haliclona* sp. (0178-22e), cf. *Lyngbya* sp. (0076-18c), and *Agelas* sp. (0049-16b), revealed at least 87 ionizable molecules, including structures of 23 proposed known [31,32,33,34,35,36,37,38,39,40,41,42,43,44,45,46,47,48,49,50,51,52] and 64 putatively new molecules (Table S2, Figure 7). For instance, the presence of tambjamine alkaloids, including tambjamines E, F, K, M, and N in *S.* cf. *signifera* (0159-22e), *C*-alkylated piperidine iminosugars, batzellasides A−C in *Haliclona* sp. (0178-22e), and terpenoid alkaloids, such as 8′-oxo-agelasin, agelasidine A, and axistatin, in *Agelas* sp. (0049-16b) extracts was suggested by their MS/MS fragmentation (Figures S2−S16). In addition, the mass spectral analysis of the *L. herbacea* EtOAc extract (0194-24c) using Orbitrap MS (positive and negative modes) electrospray ionization suggested the presence of PBDEs. In addition to the detected known compounds, the putative structures of possible new molecules **18** and **46** are proposed based on their MS/MS fragmentation (Figure S3).

**Figure 8:**
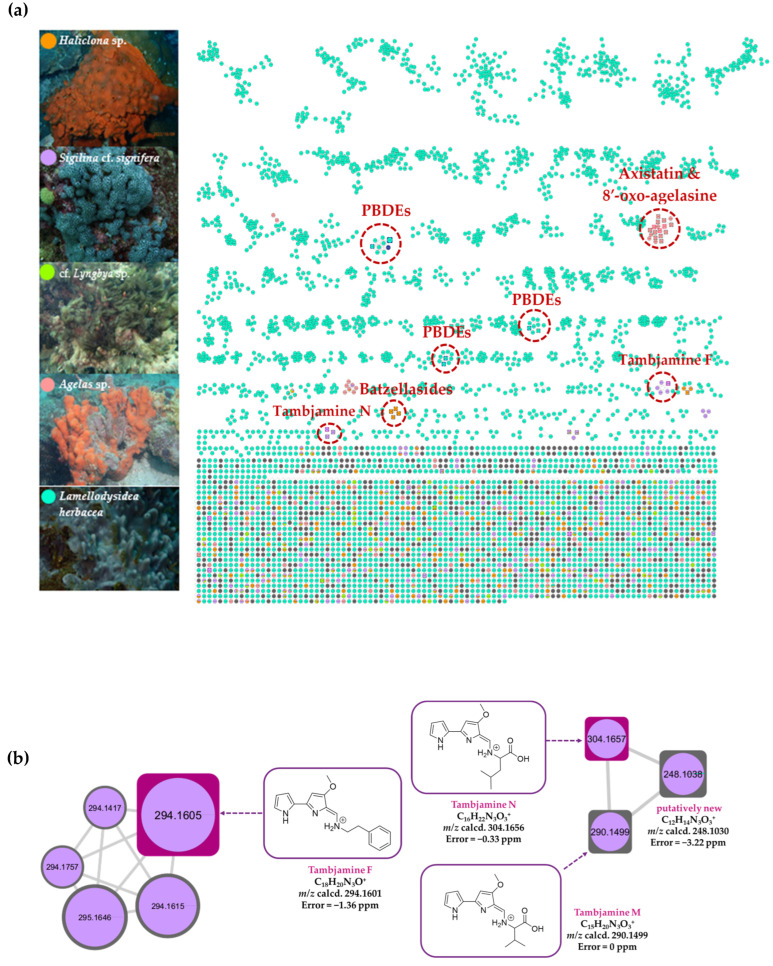

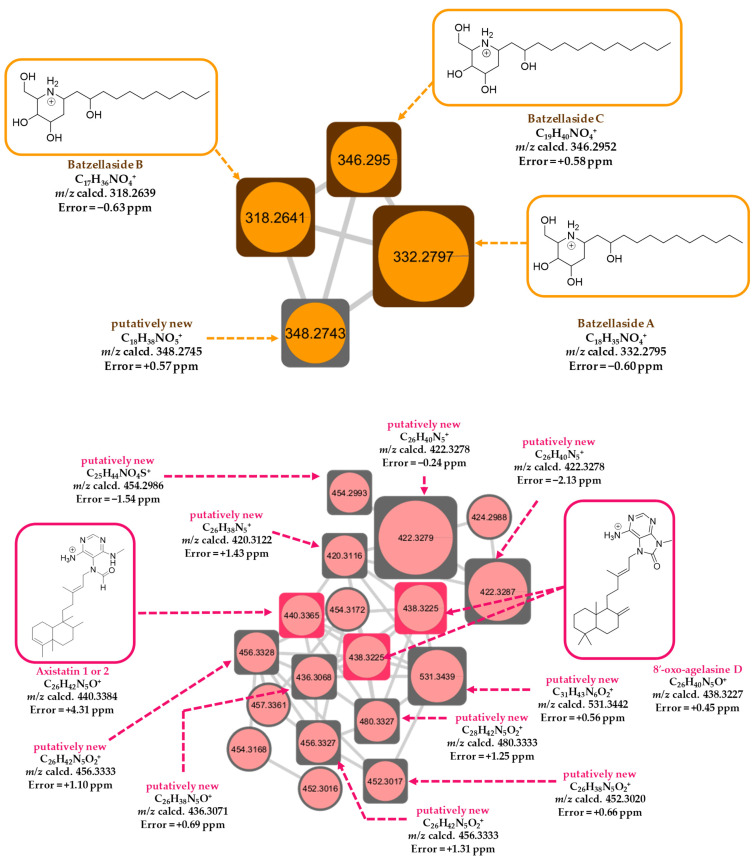

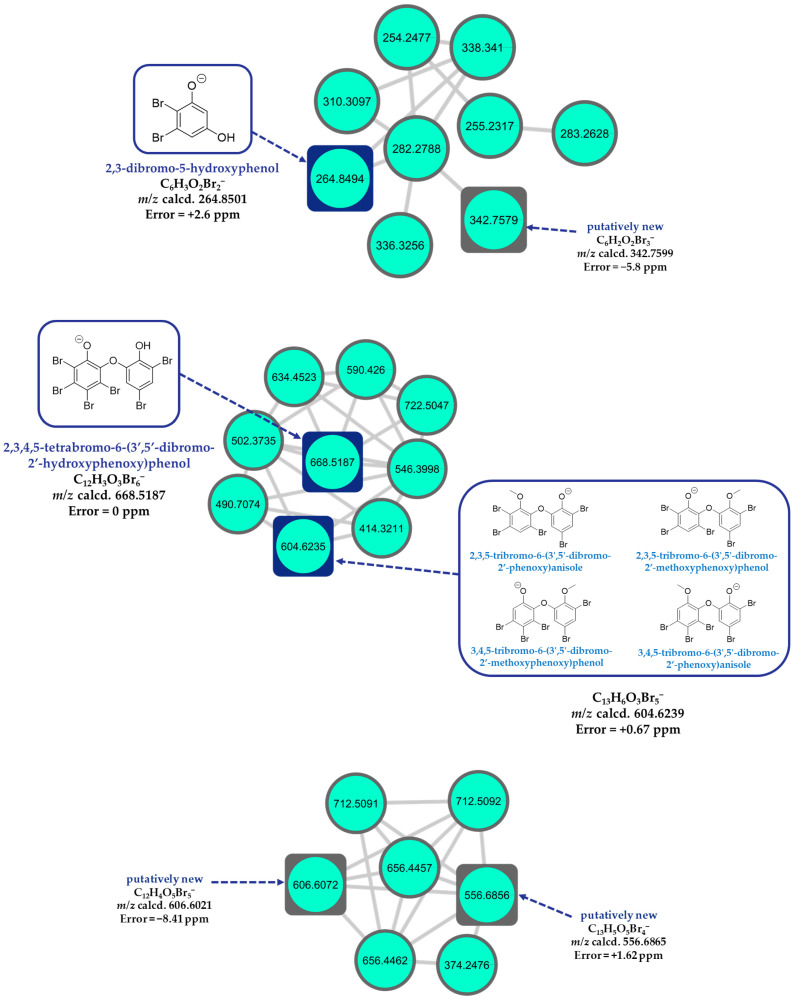

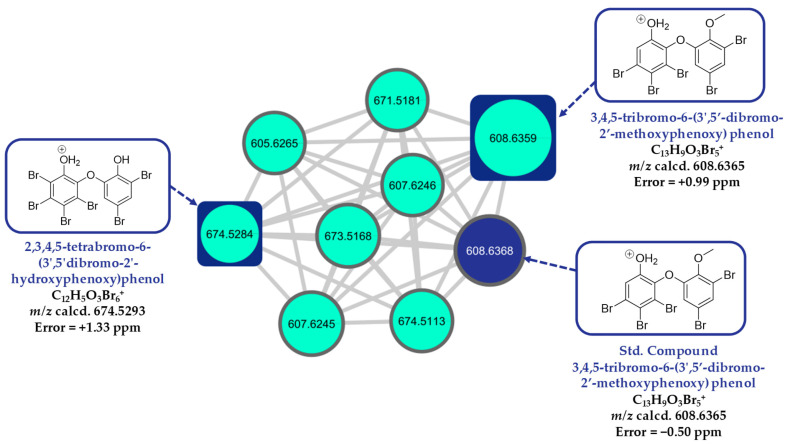
The global molecular network of the marine-derived extracts of five active samples, including EtOAc extracts of *Haliclona* sp. (0178-22e, orange), *S.* cf. *signifera* (0159-22e, purple), cf. *Lyngbya* sp. (0076-18c, light green), and *L. herbacea* (0194-24c, turquoise blue) and the H_2_O extract of *Agelas* sp. (0049-16b, pastel red) (**a**); magnification of unique molecular network clusters showing tambjamine alkaloid clusters for *S.* cf. *signifera* (0159-22e, purple), batzellaside alkaloid clusters for *Haliclona* sp. (0178-22e, orange), and agelasine alkaloid clusters for *Agelas* sp. (0049-16b, pastel red). The proposed chemical structures of known compounds related to the nodes are based on feature-based molecular networking (FBMN) MS1 and MS2 data, LC-HR-MS/MS, and the Marinlit database (**b**).

Following the chemical profiling of the five extracts, mass spectrometry-based molecular networking was introduced to establish the chemical diversity of the five marine extracts. As shown in Figure 8a, at least seven unique molecular network clusters related to the molecular families of tambjamine alkaloids in *S.* cf. *signifera*, batzellasides in *Haliclona* sp., axistatin and 8′-oxo-agelasine in *Agelas* sp., and PBDEs in *L. herbacea* extracts were observed. In addition, two organobrominated compounds, namely, 2,3,4,5-tetrabromo-6-(3′,5′-dibromo-2′-hydroxy-phenoxy)phenol and 3,4,5-tribromo-6-(3′,5′-dibromo-2′-methoxyphenoxy)phenol (Figure 8b), were confirmed through molecular networking using an internal standard [44,53].

Interestingly, *S.* cf. *signifera* (0159-22e) and *L. herbacea* (0194-24c) extracts, containing tambjamine alkaloids and PBDEs, respectively, had the most potent anti-biofouling and anti-biocorrosion activities at lower concentrations. The PBDEs and tambjamine alkaloids are known to possess antifouling activity [14,54], and they could represent new classes of eco-friendly anti-QS and anti-biocorrosion agents. Furthermore, the structural features of a commercially available metal-free antifouling agent, Econea^®^, containing arene and bromine-containing pyrrole, can be found in tambjamines and PBDEs, suggesting that these functional groups could be important for anti-biofouling activity.

The bacterial red pigment prodigiosin, structurally related to tambjamines, was reported to have quorum-sensing-inhibitory activity against *Acinetobacter baumannii* [55]. In addition, in silico analysis revealed potential interactions between AbaI and AbaR, two major QS regulators in *A. baumannii*, and prodigiosin, which interfered with biofilm formation [55]. A recent study suggested that prodigiosin could be a potential treatment for drug-resistant *P aeruginosa* and chronic biofilm infections due to the molecule’s ability to inhibit biofilm formation and prevent the production of pyocyanin and extracellular polysaccharides in vitro in the pathogenic bacterium [56].

Due to the overall structural similarities between tambjamines and homoserine lactone-based autoinducers, e.g., *N*-3-oxo-dodecanoyl-L-homoserine lactone, of *P. aeruginosa*, the former molecules could interfere with the bacterial quorum-sensing system via their interaction with the QS transcriptional activator protein LasR. Preliminary molecular docking (SwissDock based on Attracting Cavities method) [57,58] simulation using the X-ray structure of the *P. aeruginosa* LasR ligand-binding domain (LBD) (PDB ID: 2UV0) with tambjamines F—a major ion in the molecular network—M, and N (Figure 8b), revealed the binding of these alkaloids within the LBD of LasR in a similar way to the native autoinducer, *N*-3-oxo-dodecanoyl-L-homoserine lactone (Figure S23). Specifically, the terminal pyrrole moiety in the tambjamines was found to be positioned in a similar way to the homoserine lactone unit in the natural autoinducer. Interestingly, prodigiosin was also found to dock in a similar way to the tambjamines with LasR (Figure S23).

Several synthetic imino sugar derivatives have been reported to inhibit biofilm formation in *P. aeruginosa* [59]. The imino sugar moiety is also present in the batzellasides detected in the extract of *Haliclona* sp. (0178-22e) that showed anti-QS activity (Figure 8b). Despite the structural similarity to *N*-3-oxo-dodecanoyl-L-homoserine lactone, such as the presence of a long alkyl chain, batzellaside A was not found to interact with the LBD of LasR based on molecular docking. The presence of hydroxyl groups in the piperidine ring of batzellasides could prevent the ring from binding within the hydrophobic pocket of the LBD of LasR [60]. In addition, molecular docking revealed that 8′-oxo agelasine D/axistatins, detected in *Agelas* sp. (0049-16b), did not interact with the LBD of LasR. These preliminary molecular docking results suggest that batzellasides and agelasines/axistatins could interfere with a bacterial QS system by other means. Moreover, it is possible that unidentified molecules, other than the detected compounds, as well as synergistic effects of the molecules, might be responsible for the observed activities. As such, further separation and isolation of molecules would be required to confirm the identity of the bioactive compounds responsible for the observed activities.

### 2.9. PBDEs as New Anti-QS and Anti-Biofilm Agents

**Figure 9:**
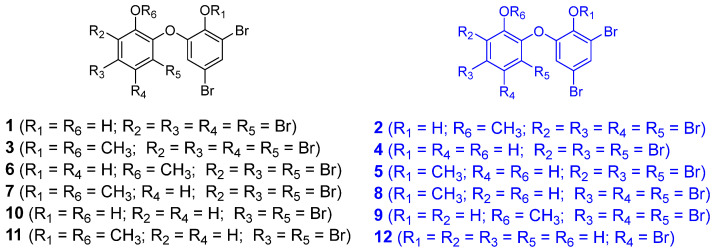
Structures of PBDEs (**1**–**12**). Structures drawn in blue color were the top six most active anti-QS PBDEs.

Further investigation of the EtOAc extract of *L. herbacea* was carried out due to its potent anti-biofouling and anti-biocorrosion activities. As such, twelve PBDEs, including seven natural PBDEs (**1**, **4**–**6**, **8**, **9**, **11**) derived from *L. herbacea* and five synthetic molecules (**2**, **3**, **7**, **10**, **12**) (Figure 9), were screened for anti-QS activity using the *P. aeruginosa* PAO1 *lasB*-*gfp* bioreporter strain. From the initial single-dose screening at 100 µg/mL, several compounds, including **2**, **4**–**6**, and **8**–**12**, showed anti-QS activity to some degree. The top six most active anti-QS PBDEs were **8**, **5**, **9**, **12**, **2**, and **4**. Due to the limited quantity of PBDEs, only five molecules were selected for further evaluation in the dose-dependent QS-inhibitory assay. This resulted in **8** and **5** having IC_50_ values of 15 µM and 46.2 µM, respectively. Interestingly, these two compounds differ only in the placement of one Br atom on the left ring structure. Preliminary molecular docking analysis was performed on compound **8** for possible interactions with the LBD of the *P. aeruginosa* LasR transcriptional protein. However, the results showed that **8** did not bind to the LBD of LasR, suggesting that the anti-quorum-sensing properties of PBDEs are probably due to other mechanism(s) and not the inhibition of the LasR transcriptional protein.

**Figure 10:**
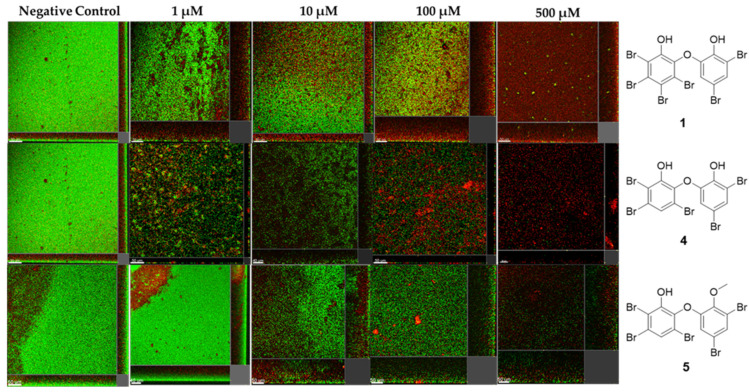
The anti-biofilm activity of compounds **1**, **4**, and **5** from the EtOAc extract of *L. herbacea*, tested on *P. aeruginosa*. Images obtained by confocal microscope. Concentrations ranged from 1 to 500 µM.

Three PBDEs, **1**, **4**, and **5**, were subsequently selected for the assessment of anti-biofilm properties. A dose-dependent study was conducted, where *P. aeruginosa* biofilms were treated with varying concentrations, 0, 1, 10, 100, and 500 µM, of these compounds. The confocal microscopy images presented in Figure 10 are representative of the biofilms formed under the influence of different concentrations of PBDEs. All three compounds demonstrated a dose-dependent inhibition of biofilm formation. Specifically, **4** and **5** treatments resulted in a marked reduction in the amount of surface-attached biofilm, indicating their strong anti-biofilm effects at a concentration of 1 µM (Figure 10). Furthermore, quantitative analysis of the biovolume from the images exhibited a significant reduction in biofilm biovolume by **1** (52.73%), **4** (83.05%), and **5** (70.68%) at 1 μM. These results are significant, as the anti-QS and anti-biofilm activities of PBDEs have not been reported. These compounds could, therefore, potentially be developed as new anti-infective agents to control *P. aeruginosa* infections and biofilm formation via QS interference.

In addition to the five most active extracts featured in the study, several other marine extracts that were not selected due to “negative” or less promising results are worth pursuing in future studies. For instance, the EtOAc extracts of several sponge species, including *Achanthostrongylophora ingens* (0001-22e and 0041-16b), *Niphates* sp. (0002-22e), *Agelas* sp. (0049-16b), and *Haliclona* sp. (0015-22e), showed significant brine shrimp lethality activity with LC_50_ ranging from 0.16 to 7.91 µg/mL (Table 1). Due to their significant toxicity, they were not selected for further assessment. However, these extracts could be further investigated as potential anticancer agents, as previous studies revealed a good correlation between cytotoxicity and brine shrimp lethality in marine natural products [61]. In particular, Indonesian *Achanthostrongylophora ingens* has been reported to contain manzamine derivatives and related alkaloids [62]. The discovery in this study that *A. ingens* (0001-22e and 0041-16b) has significant toxicity, with an average LC_50_ value of 0.46 μg/mL, could suggest that these samples contain cytotoxic compounds and warrant further chemical investigation. In addition, H_2_O extracts of *A. ingens* (0001-22e and 0041-16b), *Niphates* sp. (0002-22e), *Agelas* sp. (0049-16b), *Clathria* sp., *Plakortis* sp., and *Haliclona* sp. (0015-22e), exhibited antibacterial activity against *S. aureus* and *P. aeruginosa* (Table 1). As aqueous-derived extracts are typically not pursued extensively as compared to organic extracts, these antibacterial H_2_O extracts could represent potential sources of new antibiotics, and their observed biological activities merit further investigation.

This study comprehensively explored the biological and chemical activities of Indonesian marine organisms, identifying promising compounds for anti-quorum-sensing, anti-biofilm, anti-biofouling, and anti-biocorrosion applications. Bioassay results highlighted five active extracts, particularly from the marine tunicate *Sigilina* cf. *signifera* and the sponge *Lamellodysidea herbacea*. These extracts demonstrated significant efficacy against microbial communication, biofilm formation, biofouling of mussels, and biocorrosion of materials. Chemical analyses of the bioactive extracts, including advanced techniques, such as LC-HR-MS/MS and molecular networking, revealed the presence of both known and new molecules, notably tambjamines and PBDEs, which were possibly linked to the observed bioactivities.

The isolation and structural elucidation of these bioactive compounds are currently ongoing to enable a better understanding of their mechanisms. Future work will also focus on scaling up the production of the most promising compounds and testing their environmental safety to ensure sustainable applications. This study underscores the significant potential of marine natural products in creating eco-friendly solutions for maritime challenges, setting a foundation for continued interdisciplinary research and development.

## 3. Materials and Methods

### 3.1. Biological Material

Marine specimens were collected from Indonesian waters, including Banten (BTN), Jakarta (JSCR), Lampung (LPG), South Sulawesi (SSW), and Southwest Papua (SWP), using SCUBA. These marine specimens consisted of macro- and microorganisms, such as sponges, cnidarians, tunicates, bryozoans, mollusks, algae, marine-sourced bacteria, marine-sourced fungi, and cyanobacteria. The five selected bioactive extracts explored in detail in this study were obtained from cf. *Lyngbya* sp. (0076-18c) from BTN, *L. herbacea* (0194-24c) from BTN, *Agelas* sp. (0049-16b) from JSCR, and *Haliclona* sp. (0178-22e) and *S.* cf. *signifera* (0159-22e) from SWP.

Fresh samples with weights ranging from 100 to 1000 g were either frozen at –20 °C or stored in 96% EtOH before the workup. For marine microbial isolation, a portion of each fresh specimen was cut and homogenized with sterile natural seawater in a microtube. Each diluted liquid was spread on plates of nutrient agar (NA) for marine bacteria and potato dextrose agar (PDA) for marine fungi and cultivated at room temperature. The microorganisms were transferred to the laboratory for further isolation work. After cultivation at 23 °C for 24 h using trypticase soy agar (TSA) media, a single colony was repeatedly transferred onto the same agar medium to obtain the pure isolate of the bacterium. Meanwhile, PDA was used to obtain the pure isolates of marine fungi. The incubation for marine fungi was performed over 3 days.

### 3.2. Chemical Material

Twelve pure PBDEs (**1**–**12**) were obtained from isolation and synthesis described in a previous report [53].

### 3.3. Extraction and Partition

A portion of each marine sample was extracted exhaustively using MeOH and concentrated in vacuo to provide a methanolic extract. The MeOH extract was then partitioned between EtOAc and H_2_O to give EtOAc and H_2_O extracts.

### 3.4. Brine Shrimp (Artemia salina) Lethality Assay

All extracts were evaluated for toxicity against brine shrimp (*Artemia salina*) larvae [63]. The experiment was carried out in triplicate. Extracts possessing LC_50_ > 10 µg/mL were used for subsequent steps.

### 3.5. Agar-Plate Diffusion Assay

Paper disks were impregnated with a sample concentration of 100 µg/disk or 200 µg/disk and placed on agar plates inoculated with either *Staphylococcus aureus* or *Pseudomonas aeruginosa*. The plates were checked for inhibition zones after incubation at 37 °C for 24 h. All materials were sterilized at 121 °C for 15 min. DMSO (20%) was used to dissolve the marine samples. Oxacillin and chloramphenicol were used as positive controls for *S. aureus*, while gentamycin was used as a positive control for *P. aeruginosa*. Clear inhibition zones were measured in mm. The internal diameter of paper disks was 6 mm. The assay was performed in duplicate.

### 3.6. Biosurfactant Assay

Sample Preparation. The assay used mineral and olive oils. Each sample from EtOAc and H_2_O extracts was dissolved with 60% aqueous MeOH to give 1.42%.

Drop-Collapse Test. Oil (5 µL) was placed on a microscope slide, and a sample (8 µL) was added to the surface of the oil. The drop size and shape were observed for one minute. The experiment was run in triplicate. The result was positive when the drop was collapsed or flattened, whereas the beaded or convex drop shape was regarded as a negative result. Distilled water and 60% aqueous MeOH were used as negative controls, while 1.42% SDS was used as a positive control.

Oil Displacement Test. Distilled water (20 mL) was added to a large Petri dish, followed by adding oil (20 µL) to the surface of water. The sample (10 µL) was added to the oil surface. The oil was displaced in the presence of a biosurfactant, showing an oil-free clearance zone. The experiment was carried out in triplicate.

### 3.7. Quorum-Sensing Inhibition (QSI) Assay

Bacterial Strain and ABTGC Medium. To determine the QSI activity of the marine extracts and compounds, the *P. aeruginosa* PAO1 *las*B-*gfp* reporter strain was used. The reporter strain has its respective promoters fused to an unstable GFP (green fluorescent protein) that has a C-terminal oligopeptide extension containing the amino acids ASV [*gfp*(ASV)]; this causes the GFP to be more susceptible to degradation by housekeeping proteases and therefore to have a short half-life. As such, unstable *gfp*(ASV) allows for the monitoring of temporal QS-regulated gene expression [64]. ABTGC medium is AB minimal medium [65] containing 2.5 mg/L thiamine, supplemented with 0.2% (wt/vol) glucose and 0.2% (wt/vol) casamino acids; LB medium contains 1.0% tryptone, 0.5% yeast extract, and 1.0% NaCl, adjusted to pH 7.0. Overnight cultures were grown for 16 h at 37 °C and shaken at 150 rpm. Selective media were supplemented with gentamicin (60 mg/L) where appropriate.

*P. aeruginosa* Quorum-Sensing Inhibition Assay. Marine extracts and pure compounds were dissolved in 100% DMSO and mixed with ABTGC medium, after which they were added to the first column of wells of a 96-well microtiter plate (Nunc) to give a final concentration of 100 µg/mL in a final volume of 200 µL. The QSI assay was initially performed with a single dose at 100 µg/mL for all marine extracts, while the dose-dependent QSI assay was performed on the pure compounds. For the dose-dependent QSI assay, ABTGC (100 µL) medium was then added to the remaining wells in the plate, and serial 2-fold dilutions of the pure compounds were made by adding 100 µL of the preceding compound-containing well to the subsequent one. The final column was left without an inhibitor as a control. Next, an overnight culture of the *P. aeruginosa lasB-gfp* (ASV) strain, grown in LB medium at 37 °C with shaking, was diluted to an optical density at 600 nm (OD_600_) of 0.2, and 100 µL of the bacterial suspension was added to each well of the microtiter plate. Hence, compound concentrations ranged from 100 µg/mL to 1.563 µg/mL across the plate in a volume of 200 µL. The microtiter plate was incubated at 37 °C in a Tecan Infinite 200 Pro plate reader (Tecan Group Ltd., Männedorf, Switzerland). GFP fluorescence (excitation at 485 nm, emission at 535 nm) and cell density (OD_600_) measurements were collected at 15 min intervals for at least 16 h. The QSI assay was carried out in triplicate.

### 3.8. Anti-Biofilm Assay

Overnight cultures of GFP-labeled *P. aeruginosa* (PAO1) were grown in Luria–Bertani broth (10 g/L NaCl, 10 g/L yeast extract, and 10 g/L tryptone) at 37 °C under shaking conditions at 200 rpm. The overnight culture was diluted to an optical density at 600 nm (OD_600_) of 0.4 and added to a glass-bottomed 24-well plate with different concentrations (0–500 µM) of the pure compounds. For the marine-derived extracts, a single dose of 1000 µg/mL was used to study the anti-biofilm properties. For pure compounds, the concentrations used ranged from 1 to 500 µM. The plates were then incubated at 37 °C for 24 h for biofilm formation. The dead bacteria and eDNA in the biofilm were stained with 3 µM propidium iodide (PI; Thermo Fisher Scientific, Waltham, MA, USA) for 15 min for visualization. Three-dimensional image stacks of the surface-attached biofilm were acquired using a Carl Zeiss LSM 780 laser scanning confocal microscope (Leica Microsystems, Wetlzler, Germany) with a 20× objective. The images were processed and analyzed using Imaris 9.0 (Bitplane, South Windsor, CT, USA). The biovolumes of live bacteria within the biofilms were quantified and compared to assess the anti-biofilm properties of the extracts or the compounds. The experiment was conducted in quintuplicate.

### 3.9. Anti-Biofouling Assay

Selected samples (from both the EtOAc and H_2_O extracts) were subjected to an anti-biofouling assay using the mussel *P. indica*. An amount of 200 mL of seawater containing the sample extract was added to an aquarium (10 cm × 10 cm × 15 cm) containing 5 healthy mussel specimens. After 24 h, the adhesion of *P. indica* was observed. Two concentrations, 25 and 100 µg/mL, of samples were used. Seawater without a sample was used as a negative control, while CuSO_4_ was used as a positive control. Mussels did not show or decrease byssus adherence after 24 h, indicating that the samples had anti-biofouling activity. The experiment was carried out in duplicate with qualitative observations.

### 3.10. Anti-Biocorrosion Assay

Selected samples (from both the EtOAc and H_2_O extracts) were tested using fresh seawater in the presence of cutter blades (SK5 steel) for 3 × 24 h at room temperature. Before testing, the cutter blade was weighed. A concentration of 35 and 70 µg/mL was used, and a stirrer was placed in each vial. Stirring was maintained for each test. Observations were made on the rate of corrosion on the cutter blade. The experiment was carried out in duplicate. Seawater without an extract was used as a control. The commercial corrosion inhibitor TETRACORR CI-2915 (PT. Tetra Solusindo Kemika, Tangerang, Indonesia) was used as a positive control. The corrosion rate was calculated using the following formula:

$$\mathrm{Corrosion}\;\mathrm{rate}\;\left(\mathrm{mpy}\right)=\frac{\mathrm{weight}\;\mathrm{loss}\;\left(g\right)\times 3.45\times 10^{6}}{\mathrm{metal}\;\mathrm{density}\;\left(\frac{g}{{\mathrm{cm}}^{3}}\right)\times \mathrm{area}\;\left({\mathrm{cm}}^{2}\right)\times \mathrm{exposed}\;\mathrm{time}\;\left(h\right)}$$

### 3.11. LC-HR-MS/MS Analysis

The samples were analyzed using a UHPLC Vanquish Tandem Q Exative plus orbitrap HRMS Thermo Scientific. The Accucore C18 stationary phase (ø 2.1 mm × 100 mm, 1.5 µm, Thermo Scientific) was used, while the mobile phase was a mixture of 100% H_2_O + 0.1% formic acid (A) and 100% MeCN + 0.1% formic acid (B) with gradient elution from 5% B (0–1 min) to 5–95% B (1–25 min), 95% B (25–28 min), 5% B (28–33 min). The column temperature was maintained at 30 °C, and the injection volume was 2 µL. The conditions used for HRMS were as follows: full ms (resolution 70,000; AGC target 3 × 10^6^; maximum IT 100 ms; scan range *m*/*z* 100–1500), dd-MS^2^/dd-SIM (resolution 17,500; AGC target 1 × 10^5^; maximum IT 50 ms; loop count 5; isolation window *m*/*z* 4.0; (N)CE/stepped (N)CE nce: 18, 35, 53), dd settings (minimum AGC target 8.00 × 10^3^; peptide match preferred; exclude isotope on; dynamic exclusion 10.0 s), a sheath gas flow rate of 15, an aux gas flow rate of 3, a sweep gas flow rate of 0, a spray voltage of 3.80 kV, a capillary temperature of 320 °C, an S-lens RF level of 50.0, and an aux gas heater temp of 0 °C.

The samples were alternatively analyzed using Bruker MAXIS II (Bruker Corporation, Billerica, MA, USA) equipped with a quadrupole time-of-flight mass analyzer and a positive-mode electrospray ionization source. The MS system was coupled to an Agilent 1290 Infinity HPLC (Agilent Technologies, Santa Clara, CA, USA) equipped with a diode array detector on a Phenomenex analytical C18 column (ø 4.6 mm × 150 mm, 2.5 µm, 100 Å). The samples were eluted with a starting mobile phase of 5% MeCN/95% H_2_O (0.1% formic acid in both solvents), followed by a gradient of up to 100% MeCN for 15 min at a flow rate of 1 mL/min. The full MS parameters were as follows: a mass range of *m/z* 100–2000, a capillary voltage of 4.5 kV, nebulizer gas at 5.0 bar, dry gas at 12.0 L/min, and a dry temperature of 220 °C. The mass spectra data were obtained in data-dependent acquisition mode with electrospray source parameter settings of a capillary voltage of 4.5 kV, nebulizer gas at 5.0 bar, dry gas at 12.0 L/min, and a dry temperature of 220 °C. The full scan mass range was acquired at *m/z* 100–2000, with two precursor ions per mass peak isolation by quadrupole and MS/MS fragmentation using induced collision energy at 10.0 eV and a cycle time of 2.0 s. MS/MS experiments were conducted in auto MS/MS scan mode with no step collision. The raw data files were analyzed using Compact Data Analyst (Bruker software, Version 5.1) to provide accurate and high-resolution mass per charge of molecular ions in the sample and generate molecular formulae using Bruker Smart Formula manually.

### 3.12. MZmine 4.30 Data Pre-Processing, Feature-Based Molecular Networking, and Chemoinformatic Database Analyses

The raw LC-MS/MS data files (.d) were converted to mzML format by using MS Convert from the ProteoWizard suite [66] and then imported to MZmine 4.3.0 [67]. The noise levels for MS1 and MS2 were set to 1.0 × 10^4^ and 1.0 × 10^2^ (mass detector: exact mass), respectively. The chromatogram builder was applied with a minimum of 2 consecutive scans, a minimum intensity for consecutive scans of 1.0 × 10^4^, a minimum absolute height of 3.0 × 10^4^, and an *m/z* tolerance of *m/z* 0 or 10 ppm. For the local minimum feature resolver, the chromatographic threshold was set to 10%; search minimum in RT range, 0.1 min; minimum relative height, 10%; minimum absolute height, 1.0 × 10^4^; min ratio of peak top/edge, 1.0; peak duration range, 0.00–10.00 min; and minimum scanned data points, 3. The peak lists were deisotoped with the ^13^C isotope filter with an *m/z* tolerance of 0 or 10 ppm and an RT tolerance of 0.1 absolute min, and the most intense isotope was the representative one. Peaks were aligned across the samples using the Join aligner algorithm with an *m/z* and RT tolerance of *m/z* 0 or 10 ppm (weight of 75) and 0.1 absolute min (weight of 25), respectively. The obtained peak list was filtered using the feature list rows filter, keeping only peaks with MS2, and then exported to .mgf and .csv file formats for the feature-based molecular networking analysis. The pre-processed .mgf and .csv files were uploaded to the GNPS [68] web server https://gnps.ucsd.edu (accessed on 9 November 2024). For network creation, both the parent mass and fragment ion tolerance were set to 0.02 Da. Edges were filtered to have a cosine score above 0.6 and at least six matched peaks. The resulting network was visualized in Cytoscape 3.10.2 [69]. Chemoinformatic database analysis was mainly performed using Marinlit (https://marinlit.rsc.org/ accessed on 6 November 2024).

## 4. Conclusions

This study presents a significant advancement in the field of marine natural products targeting anti-biofouling and anticorrosion applications. It addresses a crucial gap in the existing literature by integrating bioassay-guided screening with chemical analyses to identify eco-friendly compounds from Indonesian marine organisms, including sponges and tunicates. The results from this study revealed that EtOAc extracts of the marine tunicate *S.* cf. *signifera* (0159-22e) and the marine sponge *L. herbacea* (0194-24c) demonstrated significant anti-biofouling activity against *Perna indica* and anti-biocorrosion performance with mpy 10.70 ± 0.70 for *S.* cf. *signifera* and 7.87 ± 0.86 for *L. herbacea*. In addition, chemical analyses of the active extracts, including LC-HR-MS/MS, MS-based molecular networking, and chemoinformatics, revealed the presence of tambjamines and PBDEs, which are likely contributors to the observed bioactivities. Subsequent investigations uncovered new anti-QS and anti-biofilm properties of synthetic and natural PBDEs previously derived from *L. herbacea*. Among these, **8** exhibited the most potent anti-QS activity, with an IC_50_ value of 15 µM, while **4** significantly reduced biofilm formation at a concentration of 1 µM.

## Figures and Tables

**Table 1:** Antibacterial activity of non-toxic marine-derived extracts tested against *S. aureus* and *P. aeruginosa*.

No.	Sample Code Name	Specimen	Phylum	Brine Shrimp Lethality Assay LC_50_ (µg/mL)		Antibacterial Assay 100 µg/Disk (EtOAc Extract) (φ, mm ± SD)		Antibacterial Assay 200 µg/Disk (H_2_O Extract) (φ, mm ± SD)		Location
				EtOAc Extract	H_2_O Extract	*S. aureus*	*P. aeruginosa*	*S. aureus*	*P. aeruginosa*	
1	0178-22e	*Haliclona* sp.	Porifera	51.72	8.05	1.68 ± 0.06	3.00 ± 1.41	NT	NT	SWP
2	0065-22e	*Plakortis* sp.	Porifera	>100	>100	3.49 ± 0.58	1.50 ± 0.71	3.26 ± 0.58	1.00 ± 0.00	SWP
3	0194-24c	*Lamellodysidea herbacea*	Porifera	38.35	>100	3.33 ± 0.35	NA	NA	NA	BTN
4	0036-22e	*Agelas* sp.	Porifera	>100	>100	NA	1.00 ± 0.00	2.11 ± 0.18	7.50 ± 0.71	SWP
5	0002-22e	*Niphates* sp.	Porifera	2.04	30.94	NT	NT	1.79 ± 0.59	5.75 ± 0.35	SWP
6	0001-22e	*Achanthostrongylophora ingens*	Porifera	0.76	>100	NT	NT	2.66 ± 1.16	3.50 ± 0.71	SWP
7	0021-22e	*Neopetrosia* sp.	Porifera	23.61	63.59	NT	NT	3.19 ± 0.08	2.50 ± 0.71	SWP
8	0107-18d	*Haliclona* sp.	Porifera	13.50	22.95	NT	NT	1.25 ± 0.07	2.50 ± 0.71	SSW
9	0049-16b	*Agelas* sp.	Porifera	6.95	>100	NT	NT	3.44 ± 0.08	2.50 ± 0.71	JSCR
10	0015-22e	*Haliclona* sp.	Porifera	7.91	>100	NT	NT	3.07 ± 0.06	2.25 ± 0.35	SWP
11	0027-22e	*Clathria* sp.	Porifera	>100	>100	NA	2.00 ± 0.00	2.38 ± 0.11	2.00 ± 0.00	SWP
12	0041-16b	*Achanthostrongylophora ingens*	Porifera	0.16	>100	NT	NT	3.86 ± 0.06	3.30 ± 1.41	JSCR
13	0159-22e	*Sigilina* cf. *signifera*	Chordata	41.44	>100	2.24 ± 0.02	1.50 ± 0.71	NT	NT	SWP
14	0126-22e	Unidentified	Chordata	>100	>100	NT	NT	3.09 ± 1.21	3.75 ± 1.41	SWP
15	0076-18c	cf. *Lyngbya* sp.	Cyanobacteria	>100	1.89	2.00 ± 0.53	2.50 ± 0.71	NT	NT	BTN
16	Swinholide A	-	-	0.14 ± 0.06		-	-	-	-	-
17	Paclitaxel	-	-	0.08 ± 0.01		-	-	-	-	-
18	Manzamine A	-	-	0.03 ± 0.01		-	-	-	-	-
19	Oxacilin	-	-	-		26.58 ± 0.14 (*S. aureus*) (5 μg/disk)				-
20	Chloramphenicol	-	-	-		17.82 ± 1.24 (*S. aureus*) (50 μg/disk)				-
21	Gentamicin	-	-	-		11.75 ± 0.35 (*P. aeruginosa*) (1000 μg/disk)				-

Note: NA = no activity; NT = not tested; SWP = Southwest Papua; LPG = Lampung; JSCR = Jakarta Special Capital Region; BTN = Banten; SSW = South Sulawesi.

**Table 2:** Biosurfactant properties of various marine-derived extracts used in the study.

No.	Sample Code Name	Specimen	Phylum	Extract	Drop-Collapse Assay		Oil Displacement Assay				Location	Biosurfactant Properties
					Average Time (s)		Mineral Oil		Olive Oil			
					Mineral Oil	Olive Oil	Average Time (s)	Average φ (cm)	Average Time (s)	Average φ (cm)		
1	0178-22e	*Haliclona* sp.	Porifera	EtOAc	1.3	13.6	1	8.6	1	9	SWP	++
2	0194-24c	*Lamellodysidea herbacea*	Porifera	EtOAc	3.3	7.6	1	8.6	1	irregular	BTN	++ ^c^
3	0036-22e	*Agelas* sp.	Porifera	H_2_O	2	13.6	-	-	-	-	SWP	++
4	0002-22e	*Niphates* sp.	Porifera	H_2_O	1.3	1.6	4.3	8.6	1	7.5	SWP	++
5	0021-22e	*Neopetrosia* sp.	Porifera	H_2_O	2.6	6	4	8.6	8.6	5.6	SWP	++
6	0049-16b	*Agelas* sp.	Porifera	H_2_O	3.3	14.3	6	9	1	9	JSCR	++
7	0015-22e	*Haliclona* sp.	Porifera	H_2_O	2.3	12.6	4	8.6	1	9	SWP	++
8	0159-22e	*Sigilina* cf. *signifera*	Chordata	EtOAc	3.6	4.3	3	9	1	8.1	SWP	++
9	0076-18c	cf. *Lyngbya* sp.	Cyanobacteria	EtOAc	3	12	2.6	8.6	1	8.8	BTN	++
10	1.42% SDS				6.3	17	7	6.2	1	1–5 ^a^	-	+
11	60% aqueous MeOH				22	72	62.66	7.2	1	≤1 ^b^	-	-

Note: The sample concentration used for the assay was 1.42%. The concentration of the positive control (SDS) was 1.42%, while the negative control was 60% aqueous MeOH. ^a^ Unclear spreading on olive oil was observed. However, the diameter (φ) of the control could be measured. ^b^ Irregular shape. ^c^ Biosurfactant properties were only observed in drop collapse for both oil and oil displacement for mineral-oil-only experiments.

**Table 3:** Anti-biocorrosion activity of five marine-derived extracts.

No.	Sample Code	Specimen	Phylum	Corrosion Rate (mpy)	
				35 µg/mL	70 µg/mL
1	0178-22e	* Haliclona * sp.	Porifera	14.10 ± 0.71	7.33 ± 1.19
2	0076-18c	cf. *Lyngbya* sp.	Cyanobacteria	14.85 ± 1.70	9.22 ± 1.83
3	0159-22e	* Sigilina * cf. *signifera*	Chordata	10.70 ± 1.70	7.72 ± 1.74
4	0194-24c	*Lamellodysidea herbacea*	Porifera	7.87 ± 0.86	4.64 ± 0.52
5	0049-16b	* Agelas * sp.	Porifera	12.54 ± 0.74	8.18 ± 1.70
6	Tetracorr CI-2915	positive control		13.60 ± 1.70	12.00 ± 1.70
7	Seawater	negative control		15.60 ± 2.12	

## Data Availability

The original contributions presented in this study are included in the article/Supplementary Material. Further inquiries can be directed to the corresponding author.

## Supplementary Materials

The following supporting information can be downloaded at https://www.mdpi.com/article/10.3390/molecules30061202/s1: The Supplementary Materials mainly describe the LC-HR-MS/MS analysis of the five marine-derived extracts, including the extracts of *S.* cf. *signifera* (0159-22e), *L. herbacea* (0194-24c), *Haliclona* sp. (0178-22e), cf. *Lyngbya* sp. (0076-18c), and *Agelas* sp. (0049-16b). Moreover, the Supplementary Materials also provide evidence for biosurfactant properties. Figure S1: Drop-collapse effect of Indonesian marine-derived extracts (a); positive control using 1.42% SDS in mineral oil (i), in olive oil (ii); negative control using 60% aqueous MeOH in mineral oil (iii), in olive oil (iv); drop collapse of 0178-22e in mineral oil (v), in olive oil (vi); drop collapse of 0076-18c in mineral oil (vii), in olive oil (viii); drop collapse of 0159-22e in mineral oil (ix), in olive oil (x); drop collapse of 0194-24c in mineral oil (xi), in olive oil (xii); drop collapse of 0036-22e in mineral oil (xiii), in olive oil (xiv); drop collapse of 0002-22e in mineral oil (xv), in olive oil (xvi); drop collapse of 0021-22e in mineral oil (xvii), in olive oil (xviii); drop collapse of 0049-16b in mineral oil (xix), in olive oil (xx); drop collapse of 0015-22e in mineral oil (xxi), in olive oil (xxii). The experiment was performed in triplicate. A representative photo was taken for each sample. The oil displacement effect of Indonesian marine-derived extracts (b). Figure S2. Fragment ions observed in HR-MS/MS spectra for tambjamines E, F, K, M, and N from EtOAc extract of *S.* cf. *signifera* (a); batzellasides A−C from EtOAc extract of *Haliclona* sp. (b); agelasidine A, 8′-oxo-agelasine D, axistatin 1 or 2 from H_2_O extract of *Agelas* sp. (c). Figure S3: Fragment ions observed in HR-MS/MS spectra of new compounds **18** and **46** detected in the extracts of *Haliclona* sp. and *Agelas* sp., respectively. Figure S4: ESI-MS/MS spectrum of compound **3** (tambjamine E). Figure S5: ESI-MS/MS spectrum of compound **6** (tambjamine M). Figure S6: ESI-MS/MS spectrum of compound **10** (tambjamine N). Figure S7: ESI-MS/MS spectrum of compound **11** (tambjamine K). Figure S8: ESI-MS/MS spectrum of compound **12** (tambjamine F). Figure S9: ESI-MS/MS spectrum of putative new compound **18**. Figure S10: ESI-MS/MS spectrum of compound **19** (batzellaside B). Figure S11: ESI-MS/MS spectrum of compound **20** (batzellaside A). Figure S12: ESI-MS/MS spectrum of compound **23** (batzellaside C). Figure S13: ESI-MS/MS spectrum of compound **43** ((−)-8′-oxo-agelasine D). Figure S14: ESI-MS/MS spectrum of compound **44** (agelasidine A). Figure S15: ESI-MS/MS spectrum of putative new compound **46**. Figure S16: ESI-MS/MS spectrum of **51** (axistatin 1 or 2). Figure S17: ESI-MS/MS spectrum of compound **73** (2,3-dibromo-5-hydroxyphenol). Figure S18: ESI-MS/MS spectrum of compound **81** (2,5-dibromo-6-(3′,5′-dibromo-2′-hydroxyphenoxy)phenol or 2,4,5-tribromo-6-(5′-bromo-2′-hydroxyphenoxy)phenol). Figure S19: ESI-MS/MS spectrum of compound **83** (2,3,4-tribromo-6-(3′,5′-dibromo-2′-hydroxyphenoxy)phenol or 2,4,5-tribromo-6-(3′,5′-dibromo-2′-hydroxyphenoxy)phenol or 2,3,5-tribromo-6-(3′,5′-dibromo-2′-hydroxyphenoxy)phenol or 3,4,5-tribromo-6-(3′,5′-dibromo-2′-hydroxyphenoxy) phenol). Figure S20: ESI-MS/MS spectrum of compound **84** (2,3,4,5-tetrabromo-6-(3′,5′-dibromo-2′-hydroxyphenoxy)phenol). Figure S21: ESI-MS/MS spectrum of compound **85** (3,5,6-tribromo-2-(2-bromophenoxy)phenol or 3,4,5-tribromo-2-(2-bromophenoxy)phenol or 3,4,6-tribromo-2-(2-bromophenoxy)phenol or 5,6-dibromo-2-(2,4-dibromophenoxy)phenol or 4,6-dibromo-2-(2,4′-dibromo phenoxy)phenol or 3,6-dibromo-2-(2,4′-dibromophenoxy)phenol or 3,4-dibromo-2-(2,4′-dibromophenoxy)phenol or 3,5-dibromo-2-(2,4′-dibromo phenoxy)phenol). Figure S22: ESI-MS/MS spectrum of compound 87 (2,3,5-tribromo-6-(3′,5′-dibromo-2′-phenoxy)anisole or 2,3,5-tribromo-6-(3′,5′-dibromo-2′-methoxyphenoxy)phenol or 3,4,5-tribromo-6-(3′,5′-dibromo-2′-methoxyphenoxy)phenol or 3,4,5-tribromo-6-(3′,5′-dibromo-2′-phenoxy)anisole. Figure S23: Molecular docking of the LasR ligand-binding domain (PBD ID: 2UV0) with the native autoinducer, N-3-oxo-dodecanoyl-L-homoserine lactone (= 3-oxo-C12-HSL) (A), and tambjamine F (B), tambjamine M (C), tambjamine N (D), and prodigiosin (E). Table S1. QS-inhibitory activity of marine-derived extracts based on *P. aeruginosa* PAO1 *lasB-gfp* bioreporter strain. Table S2. Observed *m/z* in five marine-derived extracts including EtOAc extracts of *Haliclona* sp. (0178-22e), cf. *Lyngbya* sp. (0076-22e), *S.* cf. *signifera* (0159-22e), and *L. herbacea* (0194-24c), and H_2_O extract of *Agelas* sp. (0049-16b).
