# Supplementary material for: Integrated Biological and Chemical Investigation of Indonesian Marine Organisms Targeting Anti-Quorum-Sensing, Anti-Biofilm, Anti-Biofouling, and Anti-Biocorrosion Activities
Source: Molecules. 2025 Mar 7;30(6):1202. doi: 10.3390/molecules30061202 (PMC11944283; doi:10.3390/molecules30061202)
Supplement: Supplementary file 1 [file molecules-30-01202-s001.zip › molecules-3494544-supplementary.pdf]

SUPPLEMENTARY MATERIAL

Integrated Biological and Chemical Investigation of Indonesian Marine Organisms Targeting Anti-Quorum Sensing, Anti-Biofilm, Anti-Biofouling and Anti-Biocorrosion Activities

Novriyandi Hanif [1,*], Jihan Azmi Miftah [1], Henny Dwi Yanti [2], Emmanuel Tope Oluwabusola [3], Vira Amanda Zahra [1], Nurul Farhana Salleh [4], Binu Kundukad [5], Lik Tong Tan [4], Nicole J. de Voogd [6,7], Nisa Rachmania [8], Marcel Jaspars [3], Staffan Kjelleberg [5,9,10], Dedi Noviendri [11], Anggia Murni [2] and Junichi Tanaka [12]

[1] Department of Chemistry, Faculty of Mathematics and Natural Sciences, IPB University, Bogor 16680, Indonesia; jihanazmi@apps.ipb.ac.id (J.A.M.); viraamandazahra@gmail.com (V.A.Z.)
[2] Tropical Biopharmaca Research Center, IPB University, Bogor 16128, Indonesia; dyantihenny@gmail.com (H.D.Y.); anggia_murni@apps.ipb.ac.id (A.M.)
[3] Marine Biodiscovery Centre, Department of Chemistry, University of Aberdeen, Aberdeen AB24 3FX, UK; emmanuel.oluwabusola3@abdn.ac.uk (E.T.O.); m.jaspars@abdn.ac.uk (M.J.)
[4] Natural Sciences and Science Education, National Institute of Education, Nanyang Technological University, 1 Nanyang Walk, Singapore 637616, Singapore; nie21.nfs@e.ntu.edu.sg (N.F.S.); liktong.tan@nie.edu.sg (L.T.T.)
[5] Singapore Center for Environmental Life Sciences Engineering (SCELSE), Nanyang Technological University, 60 Nanyang Drive, SBS-01N-27, Singapore 637551, Singapore; binu.kundukad@ntu.edu.sg (B.K.); laskjelleberg@ntu.edu.sg (S.K.)
[6] Naturalis Biodiversity Center, P.O. Box 9517, 2300 RA Leiden, The Netherlands; nicole.devoogd@naturalis.nl
[7] Institute of Biology (IBL), Leiden University, P.O. Box 9505, 2300 RA Leiden, The Netherlands
[8] Department of Biology, Faculty of Mathematics and Natural Sciences, IPB University, Bogor 16680, Indonesia; nrachmania@apps.ipb.ac.id
[9] School of Biological Sciences, Nanyang Technological University, 60 Nanyang Drive, Singapore 637551, Singapore
[10] School of Biological, Earth and Environmental Sciences, University of New South Wales, Sydney, NSW 2033, Australia
[11] Research Center for Pharmaceutical Ingredients and Traditional Medicine, BRIN Cibinong-Bogor, Bogor 16911, Indonesia; dedi025@brin.go.id
[12] Department of Chemistry, Biology and Marine Science, University of the Ryukyus, Okinawa 903-0213, Japan; jtanaka@sci.u-ryukyu.ac.jp
* Correspondence: nhanif@apps.ipb.ac.id; Tel.: +62-251-862-4567

Table of Contents

Title	Page
**Figure S1.** Drop-collapse effect of Indonesian marine-derived extracts (a); positive control using 1.42% SDS in mineral oil (i), in olive oil (ii); negative control using 60% aqueous MeOH in mineral oil (iii), in olive oil (iv); drop collapse of 0178-22e in mineral oil (v), in olive oil (vi); drop collapse of 0076-18c in mineral oil (vii), in olive oil (viii); drop collapse of 0159-22e in mineral oil (ix), in olive oil (x); drop collapse of 0194-24c in mineral oil (xi), in olive oil (xii); drop collapse of 0036-22e in mineral oil (xiii), in olive oil (xiv); drop collapse of 0002-22e in mineral oil (xv), in olive oil (xvi); drop collapse of 0021-22e in mineral oil (xvii), in olive oil (xviii); drop collapse of 0049-16b in mineral oil (xix), in olive oil (xx); drop collapse of 0015-22e in mineral oil (xxi), in olive oil (xxii). The experiment was performed in triplicate. Representative photo was taken for each sample. Oil displacement effect of Indonesian marine-derived extracts (b).	4
**Figure S2.** Fragment ions observed in HR-MS/MS spectra for tambjamines E, F, K, M and N from EtOAc extract of *S.* cf. *signifera* (a); batzellasides A−C from EtOAc extract of *Haliclona* sp. (b); agelasidine A, 8′-oxo-agelasine D, axistatin 1 or 2 from H_2O extract of *Agelas* sp. (c).	10
**Figure S3.** Fragment ions observed in HR-MS/MS spectra of new **18** and **46** detected in the extracts of *Haliclona* sp. and *Agelas* sp., respectively.	11
**Figure S4.** ESI-MS/MS spectrum of compound **3** (tambjamine E).	12
**Figure S5.** ESI-MS/MS spectrum of compound **6** (tambjamine M).	12
**Figure S6.** ESI-MS/MS spectrum of compound **10** (tambjamine N).	13
**Figure S7**. ESI-MS/MS spectrum of compound **11** (tambjamine K).	13
**Figure S8.** ESI-MS/MS spectrum of compound **12** (tambjamine F).	14
**Figure S9.** ESI-MS/MS spectrum of putative new compound **18**.	14
**Figure S10.** ESI-MS/MS spectrum of compound **19** (batzellaside B).	15
**Figure S11.** ESI-MS/MS spectrum of compound **20** (batzellaside A).	15
**Figure S12.** ESI-MS/MS spectrum of compound **23** (batzellaside C).	16
**Figure S13.** ESI-MS/MS spectrum of compound **43** ((−)-8′-oxo-agelasine D).	16
**Figure S14.** ESI-MS/MS spectrum of compound **44** (agelasidine A).	17
**Figure S15.** ESI-MS/MS spectrum of putative new compound **46**.	17
**Figure S16.** ESI-MS/MS spectrum of **51** (axistatin 1 or 2).	18
**Figure S17.** ESI-MS/MS spectrum of compound **73** (2,3-dibromo-5-hydroxyphenol).	18
**Figure S18.** ESI-MS/MS spectrum of compound 81 (2,5-dibromo-6-(3',5'-dibromo-2′-hydroxyphenoxy)phenol or 2,4,5-tribromo-6-(5'-bromo-2′-hydroxyphenoxy)phenol).	19
**Figure S19.** ESI-MS/MS spectrum of compound 83 (2,3,4-tribromo-6-(3',5'-dibromo-2′-hydroxyphenoxy)phenol or 2,4,5-tribromo-6-(3',5'-dibromo-2′-hydroxyphenoxy)phenol or 2,3,5-tribromo-6-(3',5'-dibromo-2′-hydroxyphenoxy)phenol or 3,4,5-tribromo-6-(3',5'-dibromo- 2′-hydroxyphenoxy)phenol).	19
**Figure S20.** ESI-MS/MS spectrum of compound 84 (2,3,4,5-tetrabromo-6-(3',5'-dibromo-2′-hydroxyphenoxy)phenol).	20

**Figure S21.** ESI-MS/MS spectrum of Compound 85 (3,5,6-tribromo-2-(2′-bromophenoxy)phenol or 3,4,5-tribromo-2-(2′-bromophenoxy)phenol or 3,4,6-tribromo-2-(2′-bromophenoxy)phenol or 5,6-dibromo-2-(2′,4′-dibromophenoxy)phenol or 4,6-dibromo-2-(2′,4'-dibromo phenoxy)phenol or 3,6-dibromo-2-(2′,4'-dibromophenoxy)phenol or 3,4-dibromo-2-(2′,4'-dibromophenoxy)phenol or 3,5-dibromo-2-(2′,4'-dibromo phenoxy)phenol).	21
**Figure S22.** ESI-MS/MS spectrum of Compound 87 (2,3,5-tribromo-6-(3',5'-dibromo-2′-phenoxy)anisole or 2,3,5-tribromo-6-(3',5'-dibromo-2′-methoxyphenoxy)phenol or 3,4,5-tribromo-6-(3',5'-dibromo-2′-methoxyphenoxy)phenol or 3,4,5-tribromo-6-(3',5'-dibromo-2′-phenoxy)anisole.	21
**Figure S23.** Molecular docking of the LasR-ligand binding domain (PBD ID: 2UV0) with the native autoinducer, N-3-oxo-dodecanoyl-L-homoserine lactone (= 3-oxo-C12-HSL) (A), tambjamine F (B), tambjamine M (C), tambjamine N (D), and prodigiosin (E).	22
**Table S1.** QS inhibitory activity of marine-derived extracts based on *P. aeruginosa* PAO1 *lasB-gfp* biomonitor strain.	24
**Table S2.** Observed *m/z* in five marine-derived extracts including EtOAc extract of *Haliclona* sp. (0178-22e), cf. *Lyngbya* sp. (0076-22e), *S.* cf. *signifera* (0159-22e), *L. herbacea* (0194-24c), and H_2O extract of *Agelas* sp. (0049-16b).	25

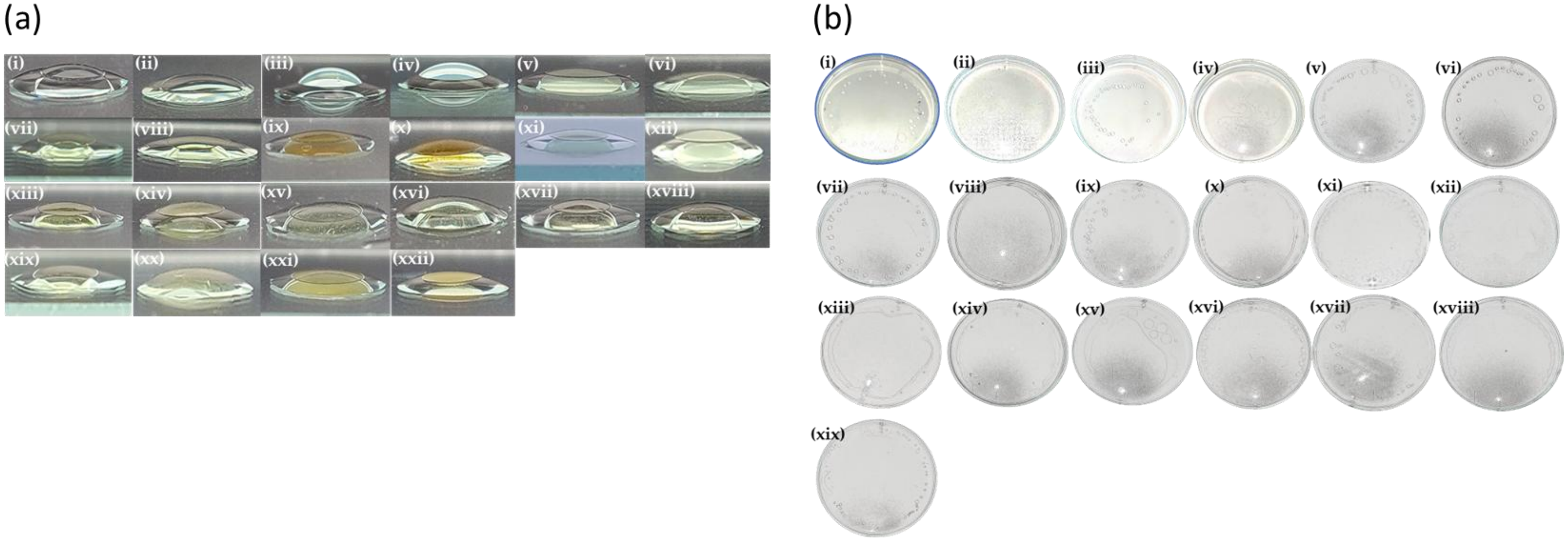

Figure S1. Drop collapsing effect of Indonesian marine-derived extracts (a); Positive control using 1.42% SDS in mineral oil (i), in olive oil (ii); negative control using 60% aqueous MeOH in mineral oil (iii), in olive oil (iv); drop collapsing of 0178-22e in mineral oil (v), in olive oil (vi); drop collapsing of 0076-18c in mineral oil (vii), in olive oil (viii); drop collapsing of 0159-22e in mineral oil (ix), in olive oil (x); drop collapsing of 0194-24c in mineral oil (xi), in olive oil (xii); drop collapsing of 0036-22e in mineral oil (xiii), in olive oil (xiv); drop collapsing of 0002-22e in mineral oil (xv), in olive oil (xvi); drop collapsing of 0021-22e in mineral oil (xvii), in olive oil (xviii); drop collapsing of 0049-16b in mineral oil (xix), in olive oil (xx); drop collapsing of 0015-22e in mineral oil (xxi), in olive oil (xxii). The experiment was performed in triplicate. Representative photo was taken for each sample. Oil displacement effect of Indonesian marine-derived extracts (b). Positive control using 1.42% SDS in mineral oil showed clear spreading (i), in olive oil showed unclear spreading (ii); negative control using 60% aqueous MeOH in mineral oil showed clear spreading (iii), in olive oil showed irregular shape (iv); oil displacement of 0178-22e in mineral oil (v), in olive oil (vi); oil displacement of 0076-18c in mineral oil (vii), in olive oil (viii); oil displacement of 0159-22e in mineral oil (ix), in olive oil (x); oil displacement of 0194-24c in mineral oil (xi); oil displacement of 0002-22e in mineral oil (xii), in olive oil (xiii); oil displacement of 0021-22e in mineral oil (xiv), in olive oil (xv); oil displacement of 0015-22e in mineral oil (xvi), in olive oil (xvii); oil displacement of 0049-16b in mineral oil (xviii), in olive oil (xix). The experiment was performed in triplicate. Representative photo was taken for each sample.

(a)

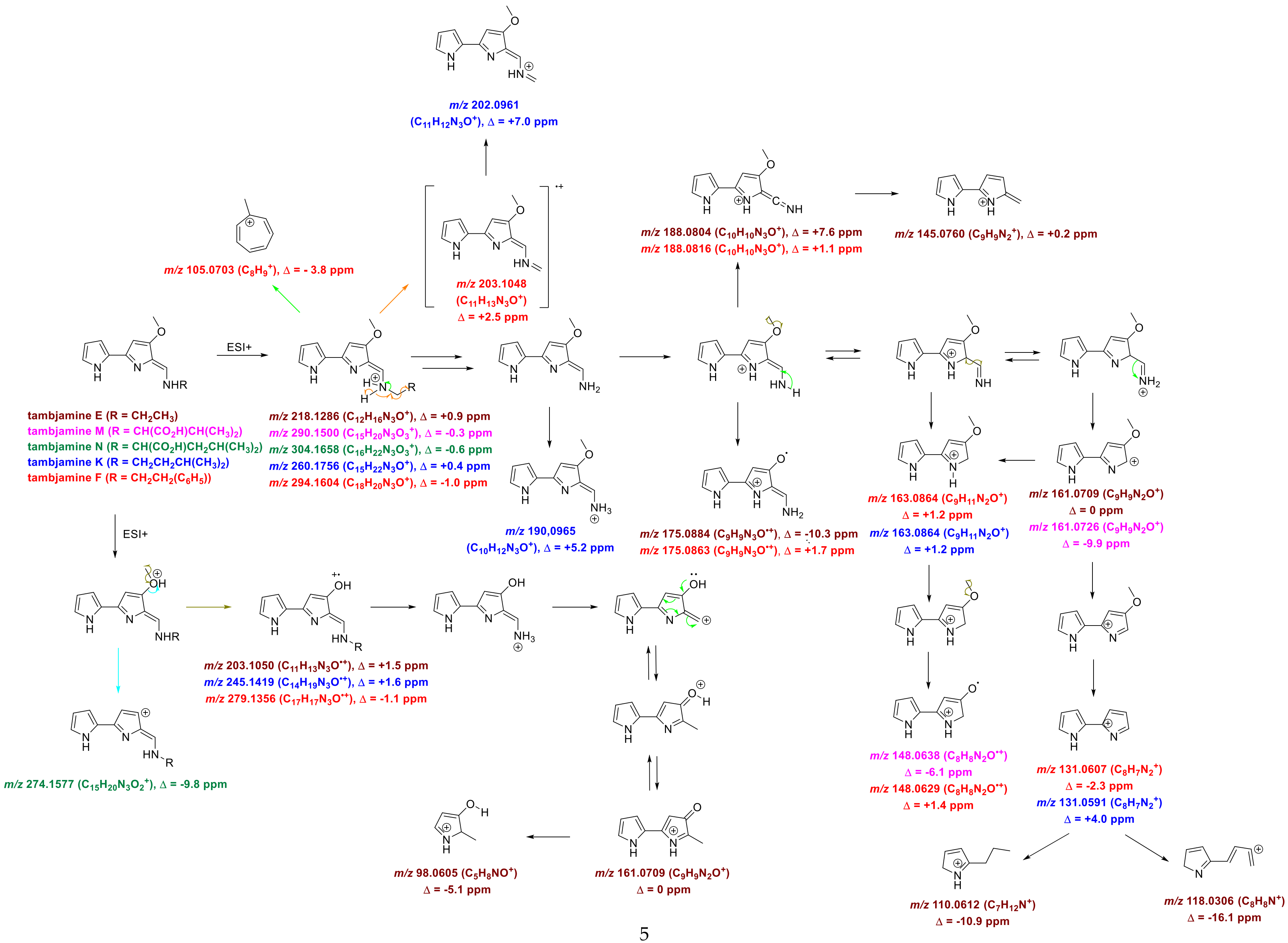

m/z 202.0961
(C11H12N3O+), Δ = +7.0 ppm
m/z 105.0703 (C8H9+), Δ = - 3.8 ppm
m/z 203.1048
(C11H13N3O+)
Δ = +2.5 ppm
m/z 188.0804 (C10H10N3O+), Δ = +7.6 ppm
m/z 188.0816 (C10H10N3O+), Δ = +1.1 ppm
m/z 145.0760 (C9H9N2+), Δ = +0.2 ppm
ESI+
tambjamine E (R = CH2CH3)
tambjamine M (R = CH(CO2H)CH(CH3)2)
tambjamine N (R = CH(CO2H)CH2CH(CH3)2)
tambjamine K (R = CH2CH2CH(CH3)2)
tambjamine F (R = CH2CH2(C6H5))
m/z 218.1286 (C12H16N3O+), Δ = +0.9 ppm
m/z 290.1500 (C15H20N3O3+), Δ = -0.3 ppm
m/z 304.1658 (C16H22N3O3+), Δ = -0.6 ppm
m/z 260.1756 (C15H22N3O+), Δ = +0.4 ppm
m/z 294.1604 (C18H20N3O+), Δ = -1.0 ppm
m/z 190,0965
(C10H12N3O+), Δ = +5.2 ppm
m/z 175.0884 (C9H9N3O•+), Δ = -10.3 ppm
m/z 175.0863 (C9H9N3O•+), Δ = +1.7 ppm
m/z 163.0864 (C9H11N2O+)
Δ = +1.2 ppm
m/z 163.0864 (C9H11N2O+)
Δ = +1.2 ppm
m/z 161.0709 (C9H9N2O+)
Δ = 0 ppm
m/z 161.0726 (C9H9N2O+)
Δ = -9.9 ppm
ESI+
m/z 203.1050 (C11H13N3O•+), Δ = +1.5 ppm
m/z 245.1419 (C14H19N3O•+), Δ = +1.6 ppm
m/z 279.1356 (C17H17N3O•+), Δ = -1.1 ppm
m/z 274.1577 (C15H20N3O2+), Δ = -9.8 ppm
m/z 148.0638 (C8H8N2O•+)
Δ = -6.1 ppm
m/z 148.0629 (C8H8N2O•+)
Δ = +1.4 ppm
m/z 131.0607 (C8H7N2+)
Δ = -2.3 ppm
m/z 131.0591 (C8H7N2+)
Δ = +4.0 ppm
m/z 98.0605 (C5H8NO+)
Δ = -5.1 ppm
m/z 161.0709 (C9H9N2O+)
Δ = 0 ppm
m/z 110.0612 (C7H12N+)
Δ = -10.9 ppm
m/z 118.0306 (C8H8N+)
Δ = -16.1 ppm

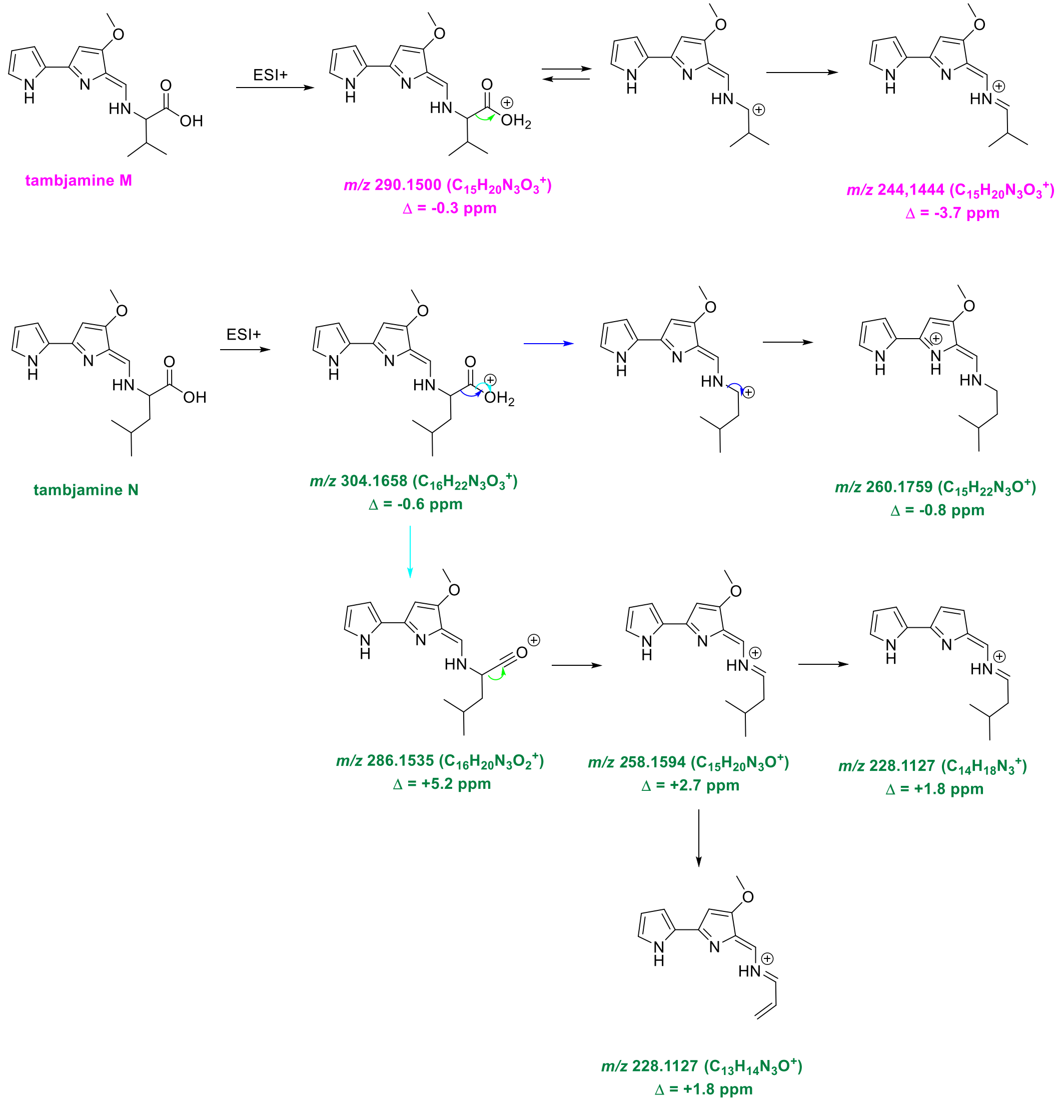
ESI+
tambjamine M
m/z 290.1500 (C15H20N3O3+)
Δ = -0.3 ppm
m/z 244,1444 (C15H20N3O3+)
Δ = -3.7 ppm
ESI+
tambjamine N
m/z 304.1658 (C16H22N3O3+)
Δ = -0.6 ppm
m/z 260.1759 (C15H22N3O+)
Δ = -0.8 ppm
m/z 286.1535 (C16H20N3O2+)
Δ = +5.2 ppm
m/z 258.1594 (C15H20N3O+)
Δ = +2.7 ppm
m/z 228.1127 (C14H18N3+)
Δ = +1.8 ppm
m/z 228.1127 (C13H14N3O+)
Δ = +1.8 ppm

(b)

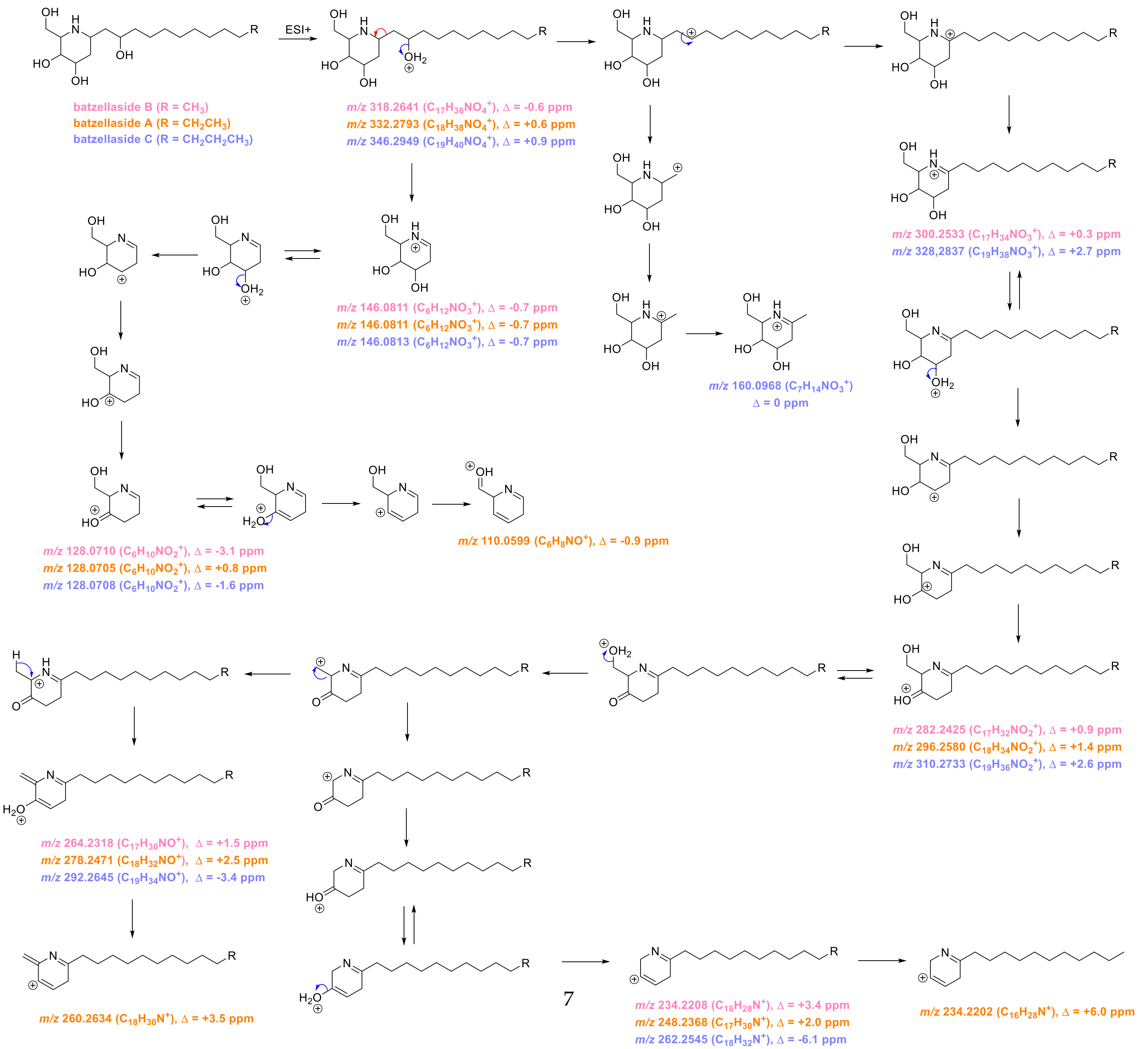
ESI+
batzellaside B (R = CH3)
batzellaside A (R = CH2CH3)
batzellaside C (R = CH2CH2CH3)
m/z 318.2641 (C17H36NO4+), Δ = -0.6 ppm
m/z 332.2793 (C18H38NO4+), Δ = +0.6 ppm
m/z 346.2949 (C19H40NO4+), Δ = +0.9 ppm
m/z 300.2533 (C17H34NO3+), Δ = +0.3 ppm
m/z 328,2837 (C19H38NO3+), Δ = +2.7 ppm
m/z 146.0811 (C6H12NO3+), Δ = -0.7 ppm
m/z 146.0811 (C6H12NO3+), Δ = -0.7 ppm
m/z 146.0813 (C6H12NO3+), Δ = -0.7 ppm
m/z 160.0968 (C7H14NO3+)
Δ = 0 ppm
m/z 128.0710 (C6H10NO2+), Δ = -3.1 ppm
m/z 128.0705 (C6H10NO2+), Δ = +0.8 ppm
m/z 128.0708 (C6H10NO2+), Δ = -1.6 ppm
m/z 110.0599 (C6H8NO+), Δ = -0.9 ppm
m/z 282.2425 (C17H32NO2+), Δ = +0.9 ppm
m/z 296.2580 (C18H34NO2+), Δ = +1.4 ppm
m/z 310.2733 (C19H36NO2+), Δ = +2.6 ppm
m/z 264.2318 (C17H30NO+), Δ = +1.5 ppm
m/z 278.2471 (C18H32NO+), Δ = +2.5 ppm
m/z 292.2645 (C19H34NO+), Δ = -3.4 ppm
m/z 260.2634 (C18H30N+), Δ = +3.5 ppm
m/z 234.2208 (C16H28N+), Δ = +3.4 ppm
m/z 248.2368 (C17H30N+), Δ = +2.0 ppm
m/z 262.2545 (C18H32N+), Δ = -6.1 ppm
m/z 234.2202 (C16H28N+), Δ = +6.0 ppm

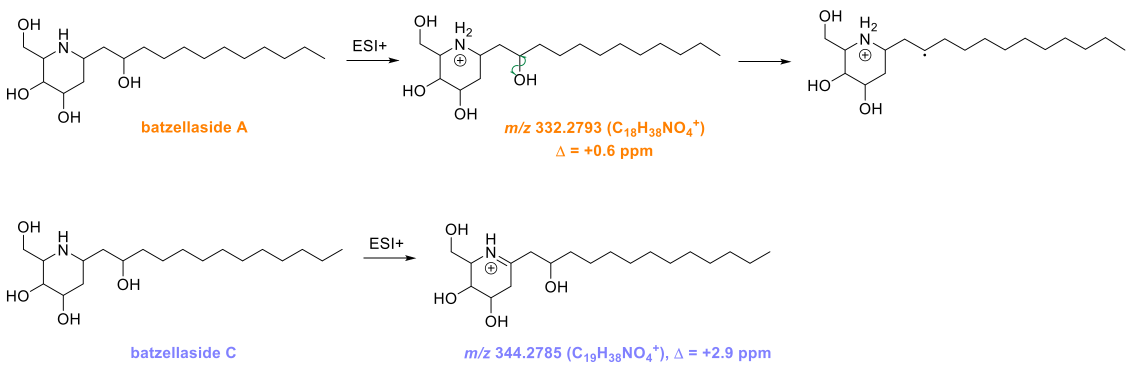
batzellaside A
ESI+
m/z 332.2793 (C18H38NO4+)
Δ = +0.6 ppm
batzellaside C
ESI+
m/z 344.2785 (C19H38NO4+), Δ = +2.9 ppm

(c)

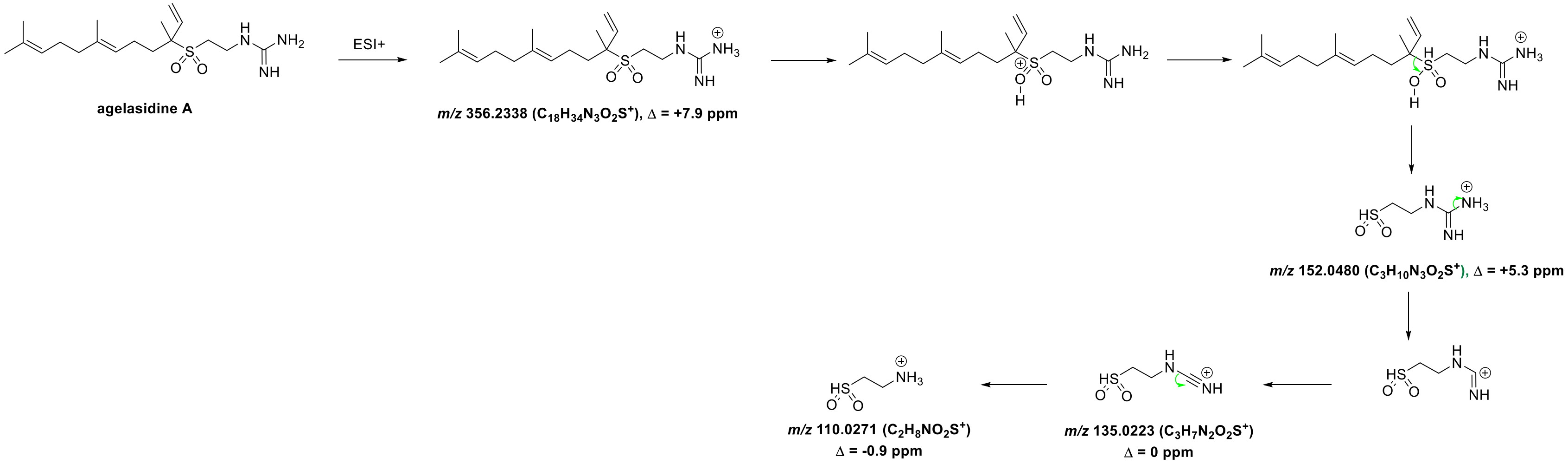
agelasidine A
ESI+
m/z 356.2338 (C18H34N3O2S+), Δ = +7.9 ppm
m/z 152.0480 (C3H10N3O2S+), Δ = +5.3 ppm
m/z 135.0223 (C3H7N2O2S+)
Δ = 0 ppm
m/z 110.0271 (C2H8NO2S+)
Δ = -0.9 ppm

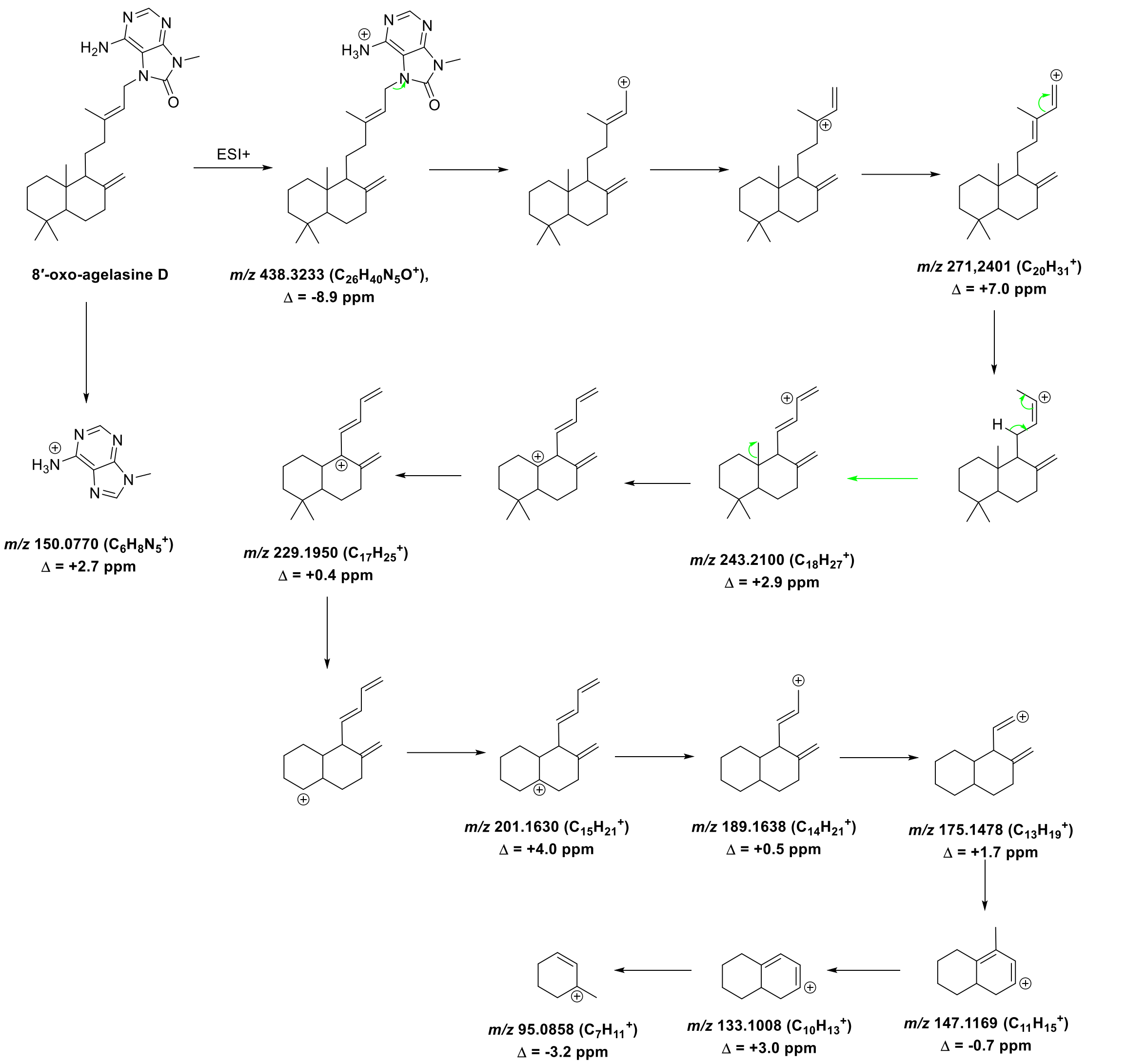
ESI+
8′-oxo-agelasine D
m/z 438.3233 (C26H40N5O+), Δ = -8.9 ppm
m/z 271,2401 (C20H31+) Δ = +7.0 ppm
m/z 150.0770 (C6H8N5+) Δ = +2.7 ppm
m/z 229.1950 (C17H25+) Δ = +0.4 ppm
m/z 243.2100 (C18H27+) Δ = +2.9 ppm
m/z 201.1630 (C15H21+) Δ = +4.0 ppm
m/z 189.1638 (C14H21+) Δ = +0.5 ppm
m/z 175.1478 (C13H19+) Δ = +1.7 ppm
m/z 95.0858 (C7H11+) Δ = -3.2 ppm
m/z 133.1008 (C10H13+) Δ = +3.0 ppm
m/z 147.1169 (C11H15+) Δ = -0.7 ppm

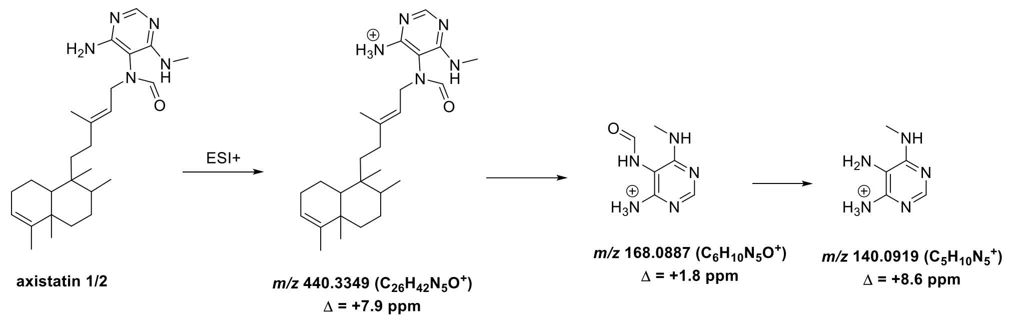

Figure S2. Fragment ions observed in HR-MS/MS spectra for tambjamines E, F, K, M and N from EtOAc extract of *S.* cf. *signifera* (a); batzellasides A–C from EtOAc extract of *Haliclona* sp. (b); agelasidine A, 8′-oxo-agelasine D, axistatin 1 and 2 from H_2O extract of *Agelas* sp. (c).

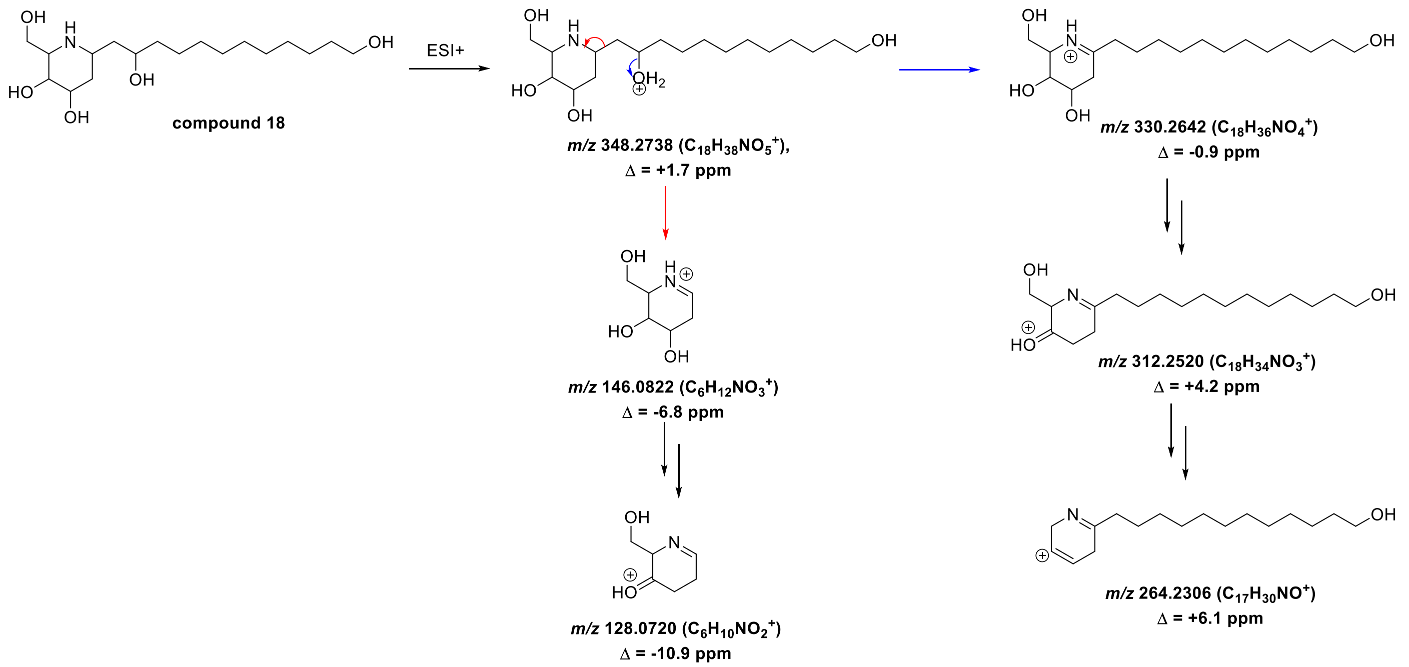

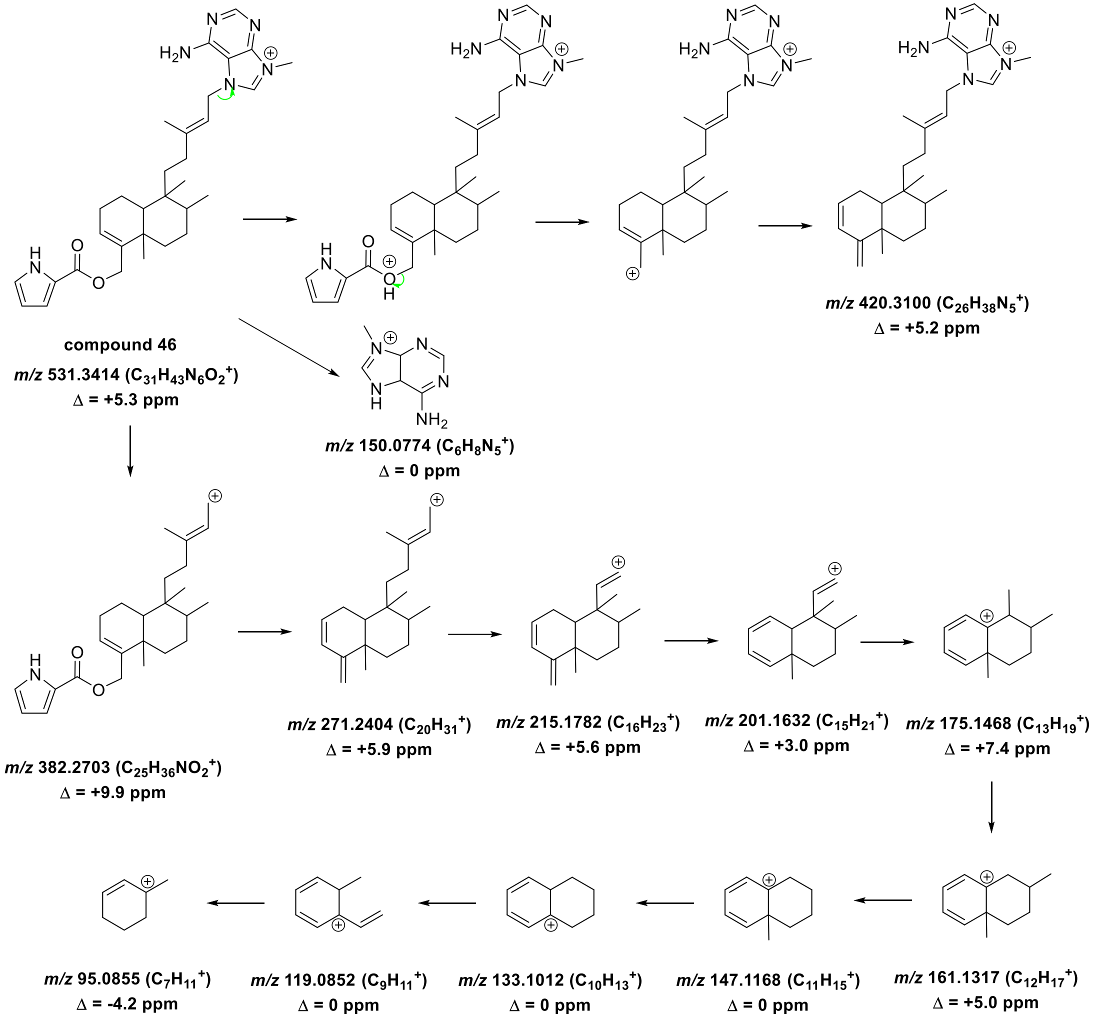

Figure S3. Fragment ions observed in HR-MS/MS spectra of new **18** and **46** detected in extracts of *Haliclona* sp. and *Agelas* sp., respectively.

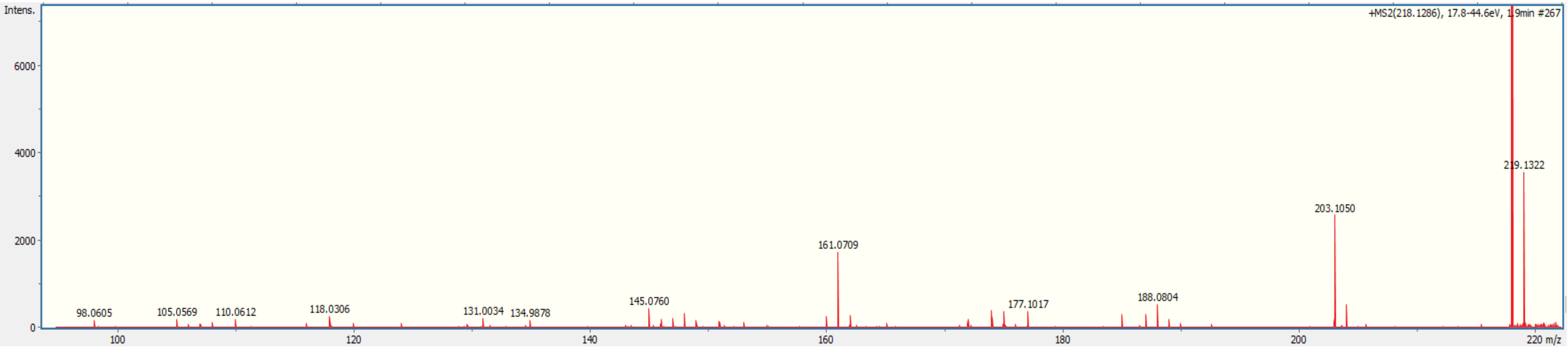

Figure S4. ESI-MS/MS spectrum of compound **3** (tambjamine E).

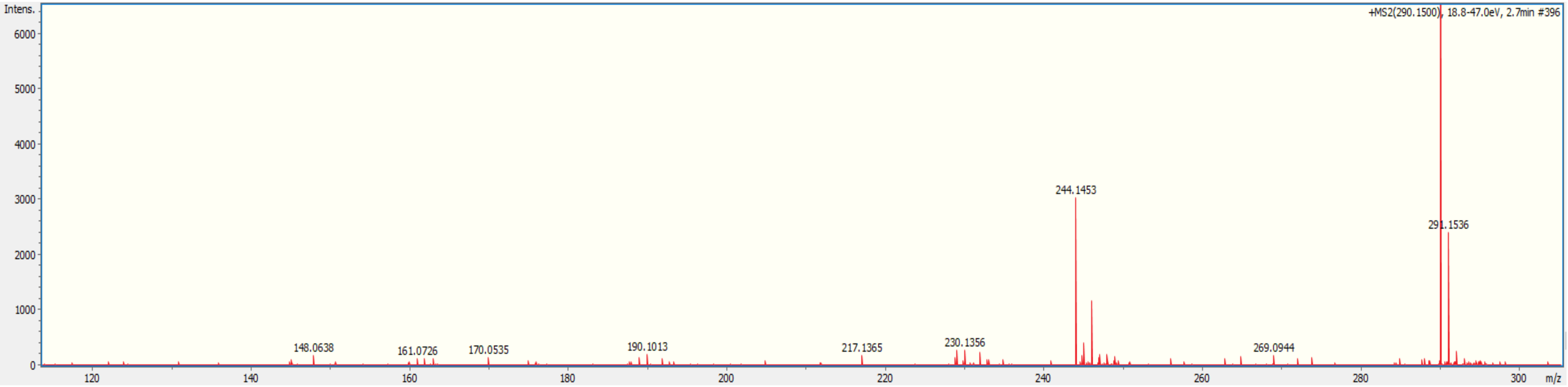

Figure S5. ESI-MS/MS spectrum of compound **6** (tambjamine M).

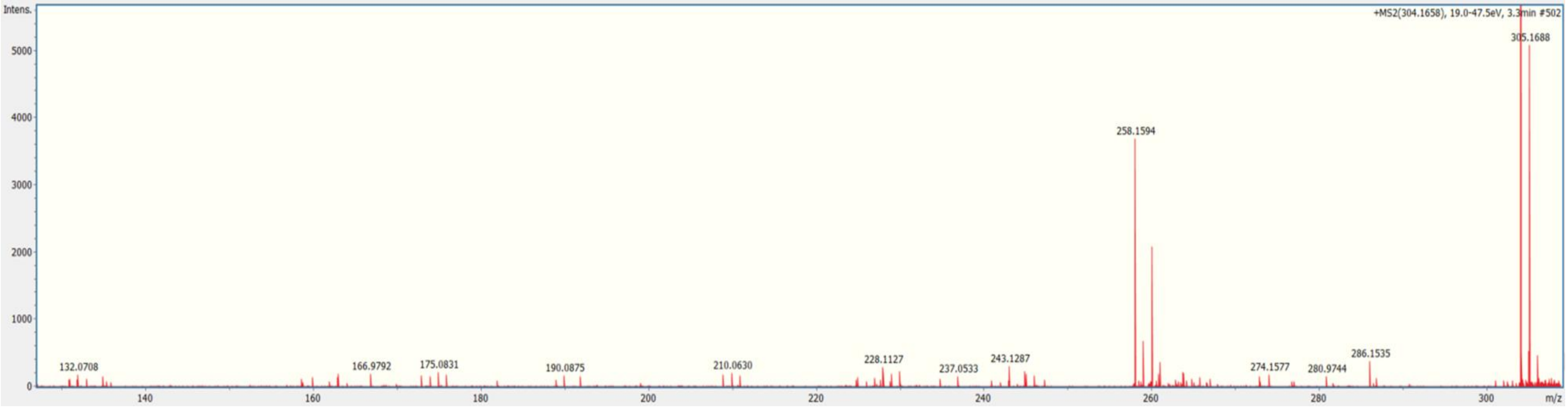

Figure S6. ESI-MS/MS spectrum of compound **10** (tambjamine N).

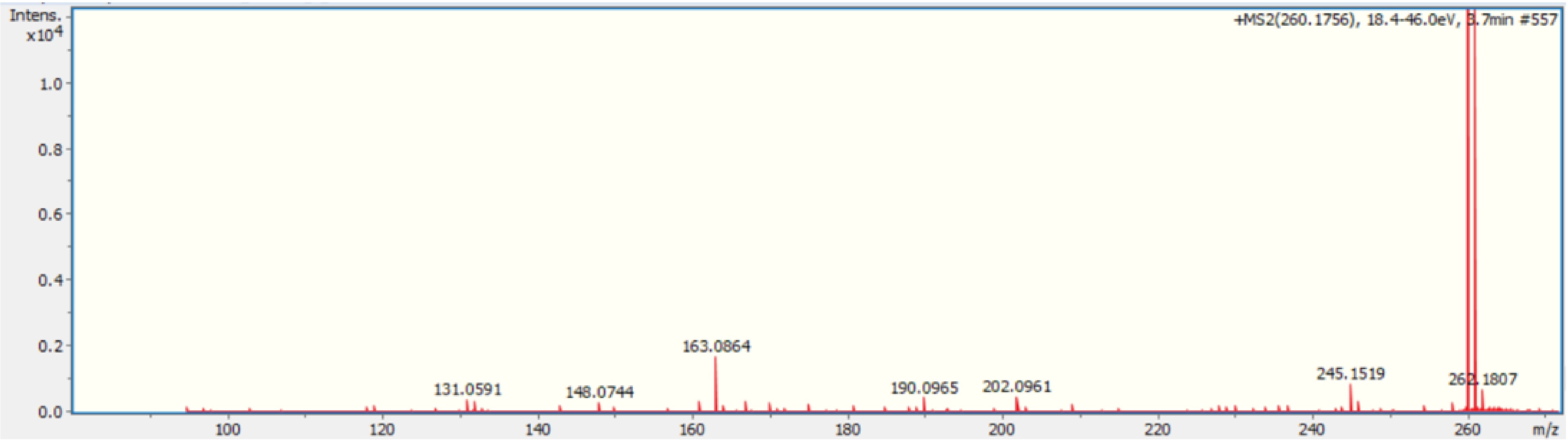

Figure S7. ESI-MS/MS spectrum of compound **11** (tambjamine K).

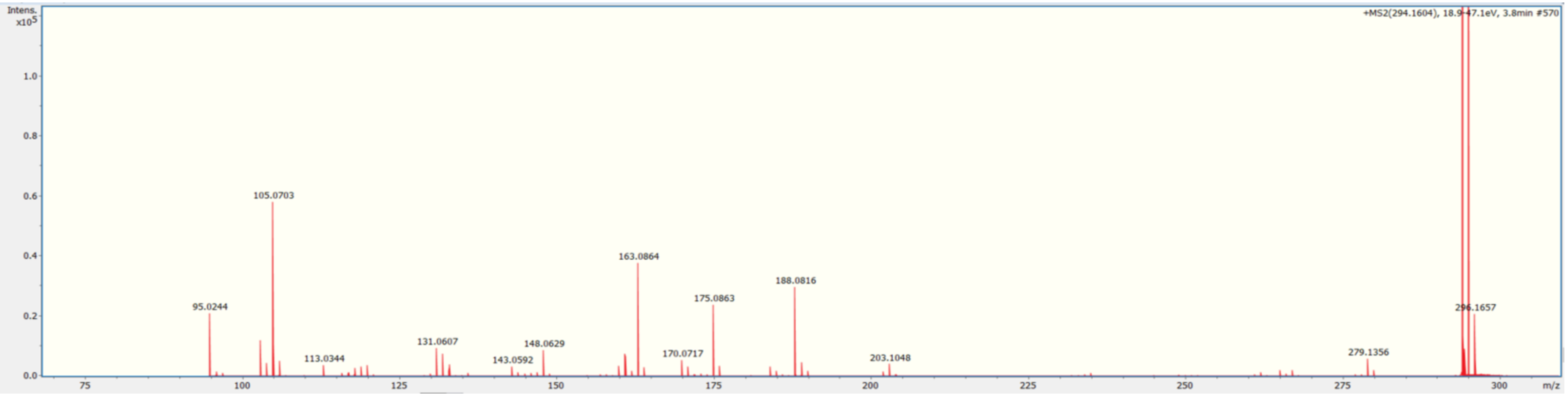

Figure S8. ESI-MS/MS spectrum of compound **12** (tambjamine F).

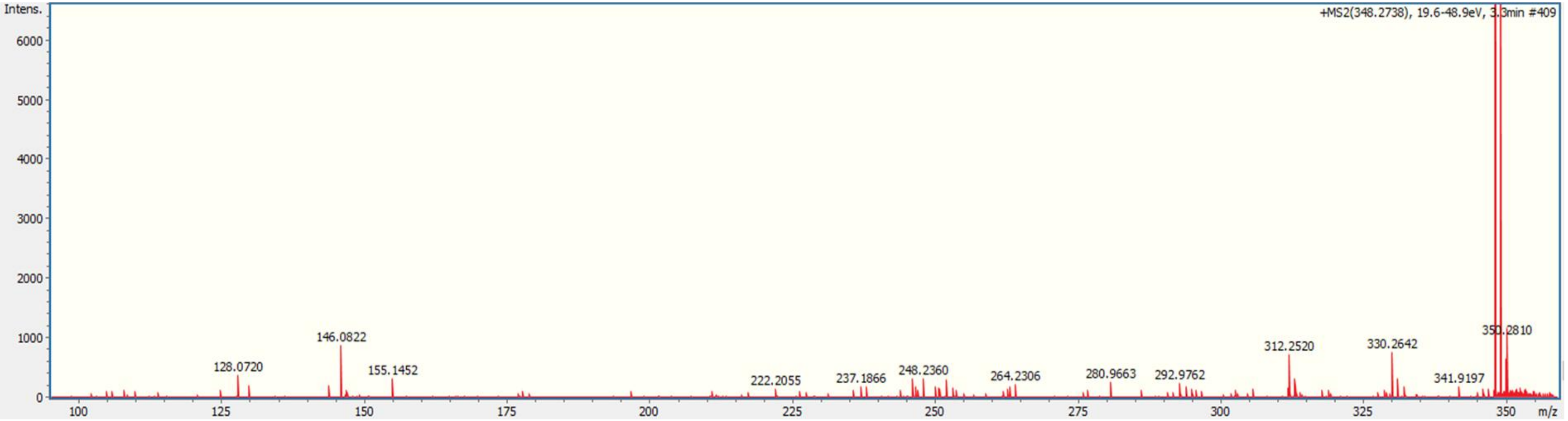

Figure S9. ESI-MS/MS spectrum of putative new compound **18**.

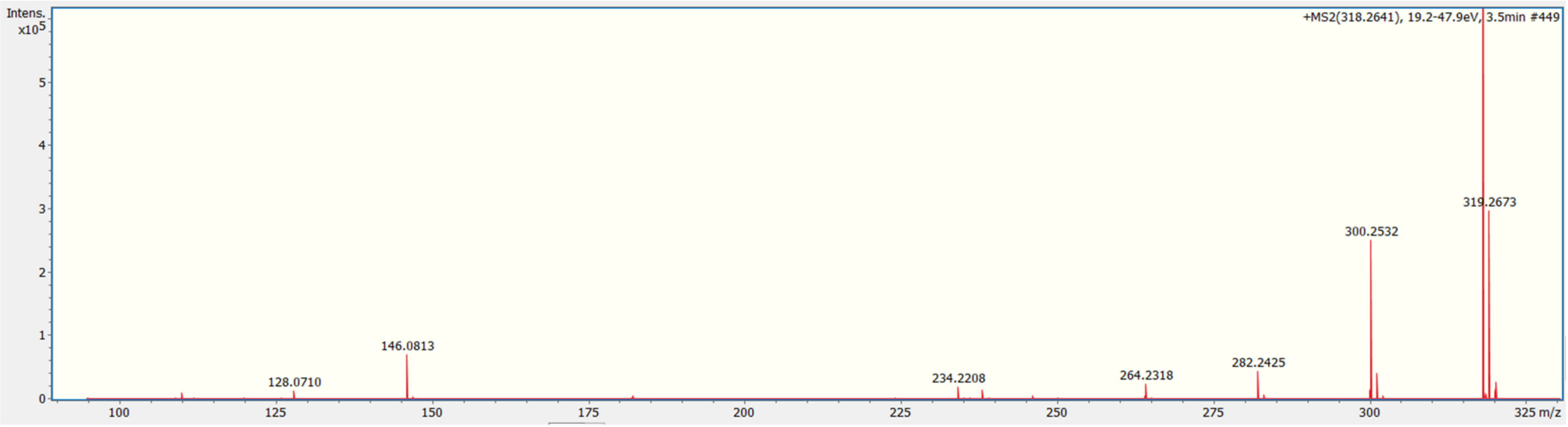

Figure S10. ESI-MS/MS spectrum of compound **19** (batzellaside B).

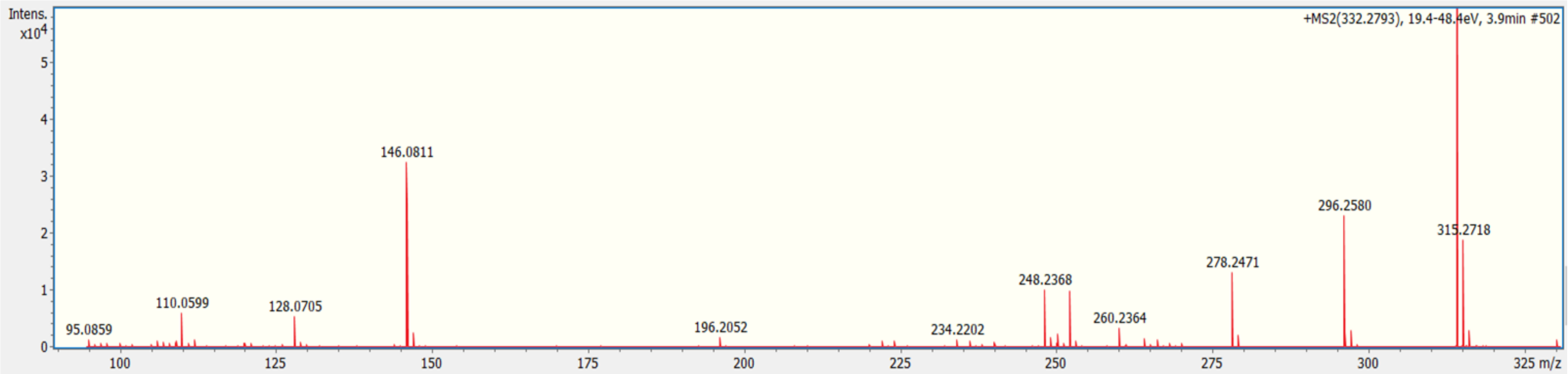

Figure S11. ESI-MS/MS spectrum of compound **20** (batzellaside A).

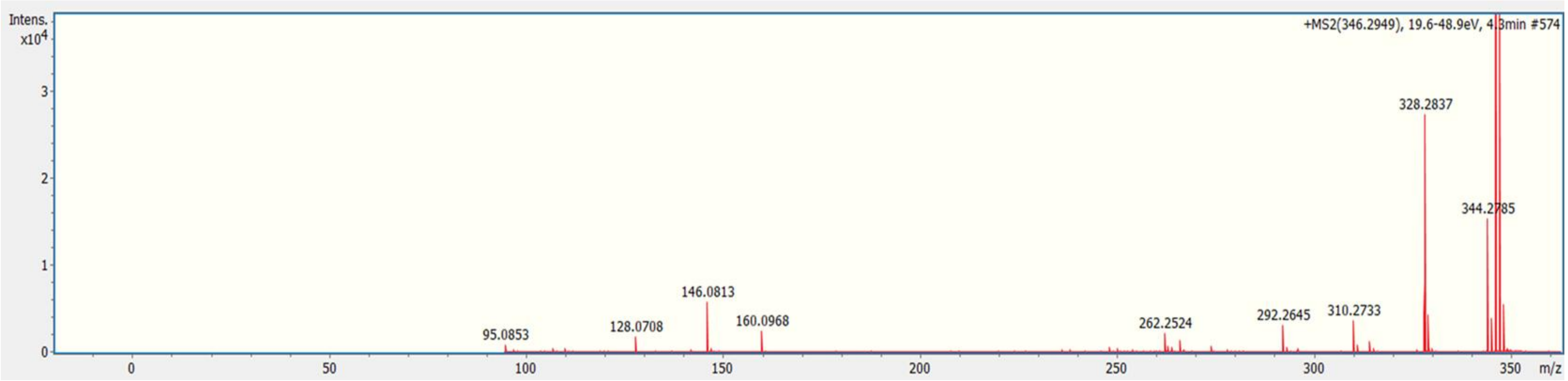

Figure S12. ESI-MS/MS spectrum of compound **23** (batzellaside C).

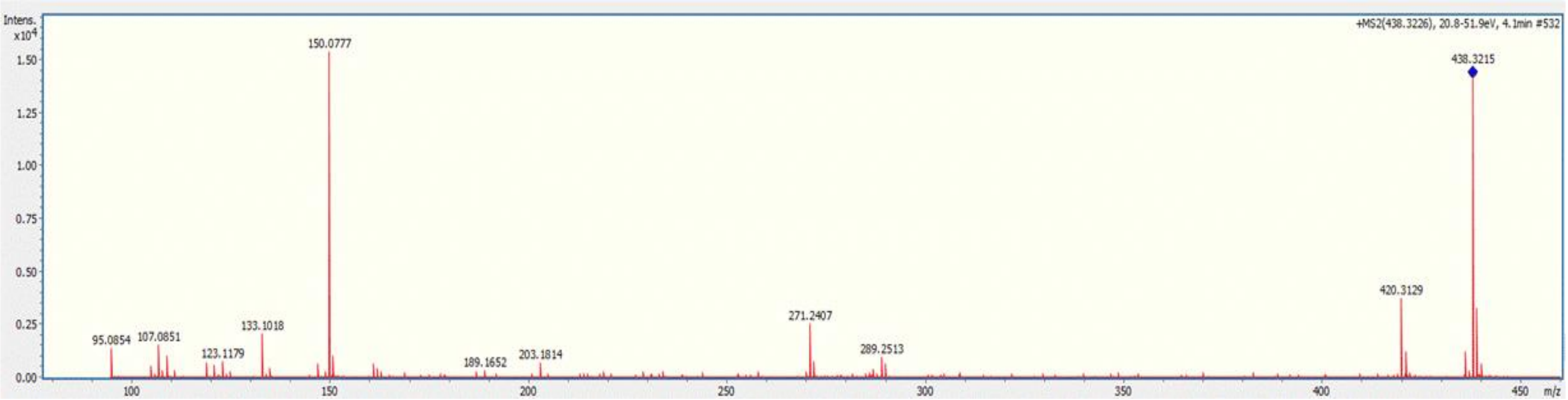

Figure S13. ESI-MS/MS spectrum of compound **43** ((−)-8′-oxo-agelasine D).

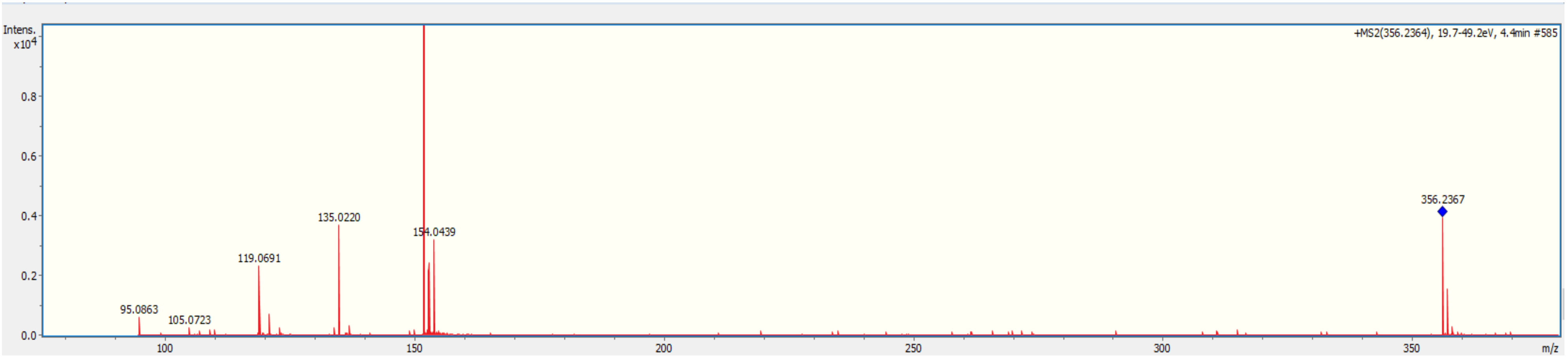

Figure S14. ESI-MS/MS spectrum of compound **44** (agelasidine A).

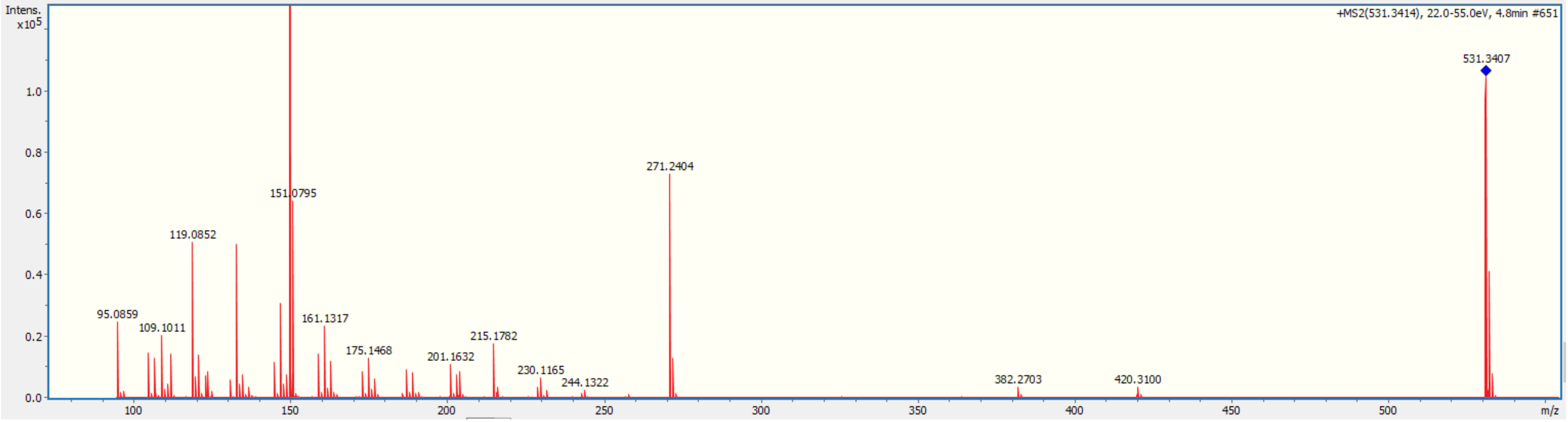

Figure S15. ESI-MS/MS spectrum of putative new compound **46**.

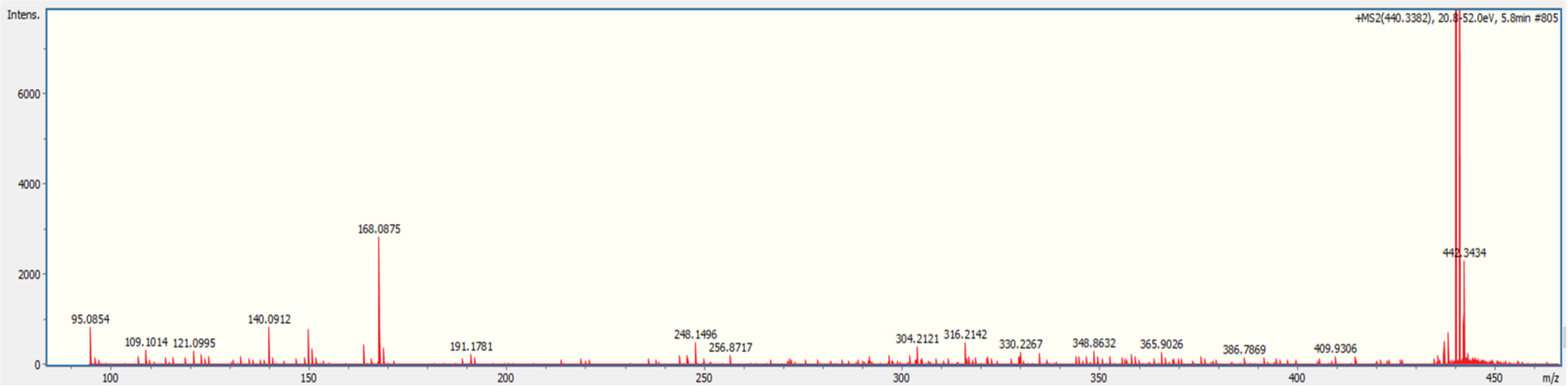

Figure S16. ESI-MS/MS spectrum of compound **51** (axistatin 1 or 2).

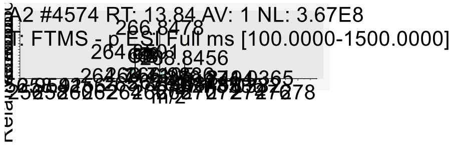

Figure S17. ESI-MS/MS spectrum of compound **73** (2,3-dibromo-5-hydroxyphenol).

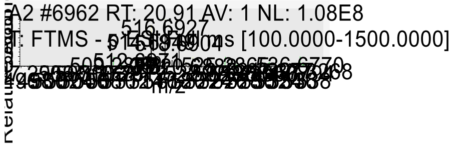

Figure S18. ESI-MS/MS spectrum of compound **81** (2,5-dibromo-6-(3',5'-dibromo-2′-hydroxyphenoxy)phenol or 2,4,5-tribromo-6-(5'-bromo-2′-hydroxyphenoxy)phenol).

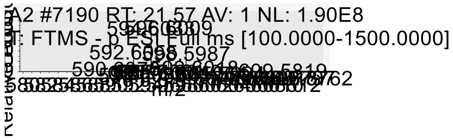

Figure S19. ESI-MS/MS spectrum of compound **83** (2,3,4-tribromo-6-(3',5'-dibromo-2′-hydroxyphenoxy)phenol or 2,4,5-tribromo-6-(3',5'-dibromo-2′-hydroxyphenoxy)phenol or 2,3,5-tribromo-6-(3',5'-dibromo-2′-hydroxyphenoxy)phenol or 3,4,5-tribromo-6-(3',5'-dibromo-2′hydroxyphenoxy)phenol).

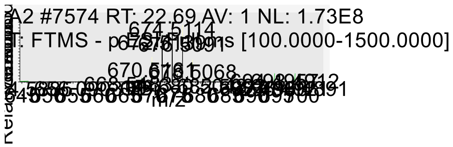

Figure S20. ESI-MS/MS spectrum of compound **84** (2,3,4,5-tetrabromo-6-(3',5'-dibromo-2′-hydroxyphenoxy)phenol)

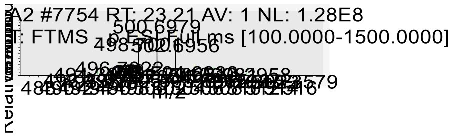

Figure S21. ESI-MS/MS spectrum of Compound **85** (3,5,6-tribromo-2-(2′-bromophenoxy)phenol or 3,4,5-tribromo-2-(2′-bromophenoxy)phenol or 3,4,6-tribromo-2-(2′-bromophenoxy)phenol or 5,6-dibromo-2-(2′,4′-dibromophenoxy)phenol or 4,6-dibromo-2-(2′,4'-dibromo phenoxy)phenol or 3,6-dibromo-2-(2′,4'-dibromophenoxy)phenol or 3,4-dibromo-2-(2′,4'-dibromophenoxy)phenol or 3,5-dibromo-2-(2′,4'-dibromo phenoxy)phenol).

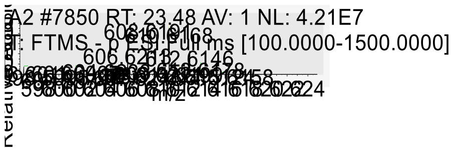

Figure S22. ESI-MS/MS spectrum of Compound **87** (2,3,5-tribromo-6-(3',5'-dibromo-2′-phenoxy)anisole or 2,3,5-tribromo-6-(3',5'-dibromo-2′-methoxyphenoxy)phenol or 3,4,5-tribromo-6-(3',5'-dibromo-2′-methoxyphenoxy)phenol or 3,4,5-tribromo-6-(3',5'-dibromo-2′-phenoxy)anisole.

(**A**) Natural ligand (3-oxo-C12-HSL)

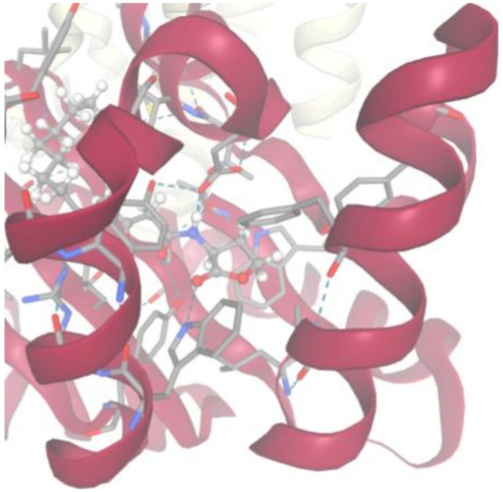

(**B**) Tambjamine F

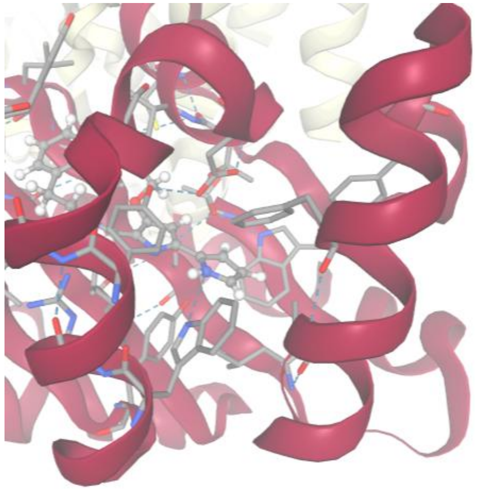

(**C**) Tambjamine M

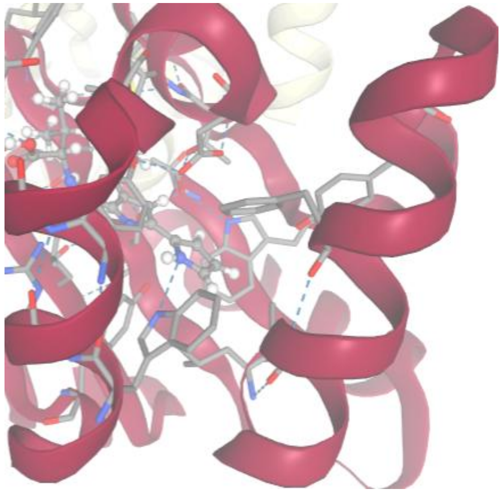

(**D**) Tambjamine N

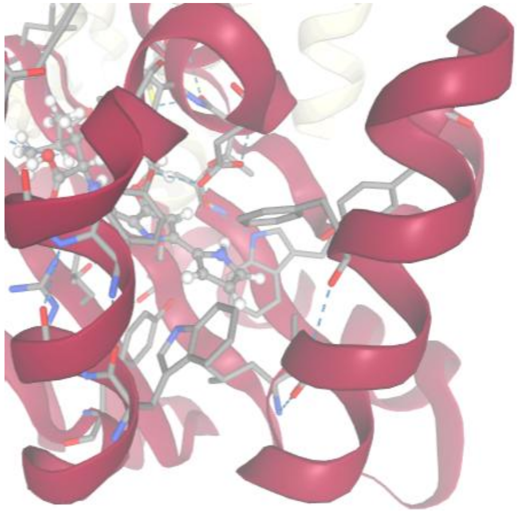

(**E**) Prodigiosin

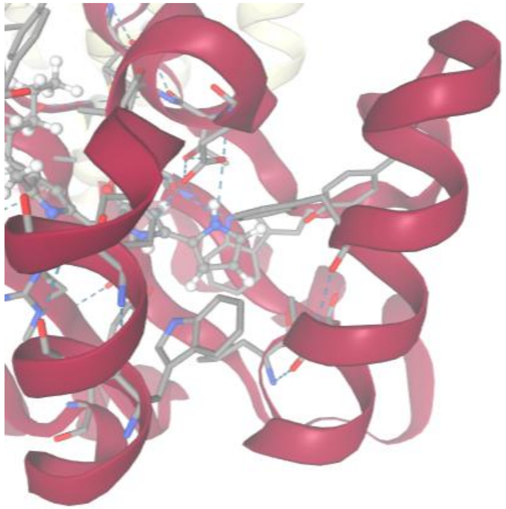

Figure S23. Molecular docking of the LasR-ligand binding domain (PBD ID: 2UV0) with the native autoinducer, N-3-oxo-dodecanoyl-L-homoserine lactone (= 3-oxo-C12-HSL) **(A)**, tambjamine F (**B**), tambjamine M (**C**), tambjamine N (**D**), and prodigiosin (**E**).

Molecular Docking

The molecular docking method applied comprises the following procedures: ligand preparation, protein selection, docking and analysis of the results. Docking was performed with the SwissDock DockingWeb Service (Available online: http://www.swissdock.ch/ (accessed on 26 February 2025). Three-dimensional structures of the autoinducer (N-3-oxododecanoyl-L-homoserine lactone), tambjamines F, M, N, prodogiosin, batzellaside A and compound 8 were either obtained from PubChem database or created on Chem3D and converted to .mol2 files using OpenBabel platform. The LasR protein structure was retrieved from the Protein Data Bank (PDB) with the reference ID (2UV0). The target ligand set was considered stable when the values of the binding free energy were lower than -7 kcal/mol. This consideration is based on docking experiments with the known X-ray structure (2UV0) complex of the autoinducer, tambjamines F, M, N, and prodigiosin resulting in binding energies values of -9.77, -8.77, -8.30, -8.46, and -8.87 kcal/mol, respectively.

Tabel S1. QS inhibitory activity of marine-derived extracts based on *P. aeruginosa* PAO1 *lasB-gfp* bioreporter strain.

No	Sample Code Name	Specimen	Phylum	Extract	Anti-QS[a]	Collection Site
1	0178-22e	*Haliclona* sp.	Porifera	EtOAc	Probably anti-QS	SWP
2	0076-18c	cf. *Lyngbya* sp.	Cyanobacteria	EtOAc	Probably anti-QS	BTN
3	0065-22e	*Plakortis* sp.	Porifera	EtOAc	Inactive	SWP
4	0159-22e	*Sigilina* cf. *signifera*	Chordata	EtOAc	Probably anti-QS	SWP
5	0194-24c	*Lamellodysidea herbacea*	Porifera	EtOAc	Probably anti-QS	BTN
6	0036-22e	*Agelas* sp.	Porifera	H_2O	Inactive	SWP
7	0002-22e	*Niphates* sp.	Porifera	H_2O	No QS but antibiotic	SWP
8	0126-22e	Unidentified	Chordata	H_2O	Inactive	SWP
9	0001-22e	*Achantostrongylophora ingens*	Porifera	H_2O	Inactive	SWP
10	0021-22e	*Neopetrosia* sp.	Porifera	H_2O	Inactive	SWP
11	0107-18d	*Haliclona* sp.	Porifera	H_2O	Inactive	SSW
12	0049-16b	*Agelas* sp.	Porifera	H_2O	Probably anti-QS	JSCR
13	0015-22e	*Haliclona* sp.	Porifera	H_2O	Inactive	SWP
14	0027-22e	*Clathria* sp.	Porifera	H_2O	Inactive	SWP
15	0041-16b	*Achantostrongylophora ingens*	Porifera	H_2O	Inactive	JSCR

Note: [a]The concentration used was 100 μg/mL.

Table S2. Observed *m/z* in five marine-derived extracts including EtOAc extract of *Haliclona* sp. (0178-22e), cf. *Lyngbya* sp. (0076-22e), *S.* cf. *signifera* (0159-22e), *L. herbacea* (0194-24c), and H_2O extract of *Agelas* sp. (0049-16b).

Compound	t_R (min)	Observed MS (*m/z*)	Formula	Calculated MS (*m/z*)	Δ (ppm)
***S.* cf. *signifera* (0159-22e)**					
Compound **1** (putatively new)	0.9	181.0719	$C_7H_9N_4O_2^+$	181.0720	−0.5
Compound **2** (putatively new)	1.8	248.1032	$C_{12}H_{14}N_3O_3^+$	248.1030	+0.8
Compound **3** (tambjamine E)	2.0	218.1286	$C_{12}H_{16}N_3O^+$	218.1288	+0.9
Compound **4** (putatively new)	2.5	308.1393	$C_{18}H_{18}N_3O_2^+$	308.1394	−0.3
Compound **5** (putatively new)	2.7	253.0818	$C_{11}H_{13}N_2O_5^+$	253.0819	−0.4
Compound **6** (tambjamine M)	2.9	290.1500	$C_{15}H_{20}N_3O_3^+$	290.1499	+0.3
Compound **7** (putatively new)	3.0	191.0813	$C_{10}H_{11}N_2O_2^+$	191.0821	−1.1
Compound **8** (putatively new)	3.0	356.1602	$C_{19}H_{22}N_3O_4^+$	356.1605	−0.8
Compound **9** (putatively new)	3.3	365.1243	$C_{19}H_{17}N_4O_4^+$	365.1244	−0.3
Compound **10** (tambjamine N)	3.4	304.1658	$C_{16}H_{22}N_3O_3^+$	304.1656	−0.7
Compound **11** (tambjamine K)	3.6	260.1756	$C_{15}H_{22}N_3O^+$	260.1757	+0.4
Compound **12** (tambjamine F)	4.0	294.1604	$C_{18}H_{20}N_3O^+$	294.1601	−1.0
Compound **13** (putatively new)	5.5	304.2998	$C_{21}H_{38}N^+$	304.2999	−0.2
Compound **14** (putatively new)	6.7	332.3314	$C_{23}H_{42}N^+$	332.3312	+0.7
Compound **15** (putatively new)	7.1	326.3781	$C_{22}H_{48}N^+$	326.3781	0
***Haliclona* sp. (0178-22e)**					
Compound **16** (putatively new)	2.2	212.1755	$C_{11}H_{22}N_3O^+$	212.1757	−0.9
Compound **17** (putatively new)	3.2	304.2483	$C_{16}H_{34}NO_4^+$	304.2482	+0.2
Compound **18** (putatively new)	3.3	348.2738	$C_{18}H_{38}NO_5^+$	348.2744	+1.7
Compound **19** (batzellaside B)	3.8	318.2641	$C_{17}H_{36}NO_4^+$	318.2639	−0.6
Compound **20** (batzellaside A)	4.1	332.2793	$C_{18}H_{38}NO_4^+$	332.2795	+0.6
Compound **21** (batzellaside A)	4.2	332.2793	$C_{18}H_{38}NO_4^+$	332.2795	+0.6
Compound **22** (batzellaside A)	4.3	332.2793	$C_{18}H_{38}NO_4^+$	332.2795	+0.6
Compound **23** (batzellaside C)	4.6	346.2949	$C_{19}H_{40}NO_4^+$	346.2952	+0.8
Compound **24** (putatively new)	5.5	280.2633	$C_{18}H_{34}NO^+$	280.2635	−0.7
Compound **25** (putatively new)	5.7	282.2788	$C_{18}H_{36}NO^+$	282.2791	−1.1
Compound **26** (putatively new)	5.9	344.3308	$C_{24}H_{42}N^+$	344.3312	−1.2
Compound **27** (putatively new)	7.1	326.3779	$C_{22}H_{48}N^+$	326.3781	−0.6
Compound **28** (putatively new)	7.6	373.2347	$C_{19}H_{29}N_6O_2^+$	373.2355	+0.3
Compound **29** (putatively new)	8.8	347.3154	$C_{20}H_{43}O_4^+$	347.3156	−0.6

Compound **30** (ceratodictyol C/D or E/F)	8.9	331.2840	$C_{19}H_{39}O_4^+$	331.2843	−0.9
Compound **31** (putatively new)	9.3	361.3307	$C_{21}H_{45}O_4^+$	361.3312	−1.4
Compound **32** (putatively new)	9.4	317.3049	$C_{19}H_{41}O_3^+$	317.3050	−0.3
Compound **33** (putatively new)	9.6	365.3026	$C_{19}H_{37}N_6O^+$	365.3032	−0.8
Compound 34 (putatively new)	9.9	375.3470	$C_{22}H_{47}O_4^+$	375.3469	+0.3
Compound **35** (putatively new)	9.9	381.2970	$C_{19}H_{37}N_6O_2^+$	381.2973	−0.7
Compound **36** (putatively new)	10.4	345.3365	$C_{21}H_{45}O_3^+$	345.3363	+0.5
***Agelas* sp. (0049-16b)**					
Compound **37** (putatively new)	3.0	456.3328	$C_{26}H_{42}N_5O_2^+$	456.3333	−1.1
Compound **38** (putatively new)	3.5	452.3020	$C_{26}H_{38}N_5O_2^+$	452.3020	0
Compound **39** ((−)-8′-oxo-agelasine D)	3.7	438.3215	$C_{26}H_{40}N_5O^+$	438.3227	+0.2
Compound **40** (putatively new)	3.8	456.3329	$C_{26}H_{42}N_5O_2^+$	456.3333	−0.9
Compound **41** (putatively new)	3.8	436.3070	$C_{26}H_{38}N_5O^+$	436.3071	−0.2
Compound **42** (putatively new)	4.0	456.3329	$C_{26}H_{42}N_5O_2^+$	456.3333	−0.9
Compound **43** ((−)-8′-oxo-agelasine D)	4.1	438.3228	$C_{26}H_{40}N_5O^+$	438.3227	+0.2
Compound **44** (agelasidine A)	4.5	356.2367	$C_{18}H_{34}N_3O_2S^+$	356.2366	+0.2
Compound **45** (putatively new)	4.5	480.3333	$C_{28}H_{42}N_5O_2^+$	480.3333	0
Compound **46** (putatively new)	4.8	531.3448	$C_{31}H_{43}N_6O_2^+$	531.3442	+1.1
Compound **47** (putatively new)	4.9	531.3448	$C_{31}H_{43}N_6O_2^+$	531.3442	+1.1
Compound **48** (putatively new)	5.1	420.3122	$C_{26}H_{38}N_5^+$	420.3122	0
Compound **49** (putatively new)	5.2	422.3293	$C_{26}H_{40}N_5^+$	422.3278	+3.5
Compound **50** (putatively new)	5.5	422.3291	$C_{26}H_{40}N_5^+$	422.3278	+3.1
Compound **51** (axistatin 1 or 2)	5.8	440.3378	$C_{26}H_{42}N_5O^+$	440.3384	−1.3
Compound **52** (putatively new)	6.5	326.3782	$C_{22}H_{48}N^+$	326.3781	+0.3
Compound **53** (putatively new)	8.2	331.2837	$C_{19}H_{39}O_4^+$	331.2843	−1.8
Compound **54** (putatively new)	8.7	454.2993	$C_{25}H_{44}NO_4S^+$	454.2986	+1.5
Compound **55** (putatively new)	8.9	454.2993	$C_{25}H_{44}NO_4S^+$	454.2986	+1.5
Compound **56** (putatively new)	9.2	381.2972	$C_{19}H_{37}N_6O_2^+$	381.2973	−0.1
Compound **57** (putatively new)	10.1	338.3415	$C_{22}H_{44}NO^+$	338.3417	−0.7
**cf. *Lyngbya* sp. (0076-18c)**					
Compound **58** (putatively new)	2.1	245.1286	$C_{14}H_{17}N_2O_2^+$	245.1285	+0.4
Compound **59** (putatively new)	5.5	304.2995	$C_{21}H_{38}N^+$	304.2999	−1.31
Compound **60** (putatively new)	6.2	332.3309	$C_{23}H_{42}N^+$	332.3312	−0.9
Compound **61** (putatively new)	6.5	326.3778	$C_{22}H_{48}N^+$	326.3781	−0.9
Compound **62** (putatively new)	8.1	331.2845	$C_{19}H_{39}O_4^+$	331.2843	+0.6
Compound **63** (putatively new)	8.1	331.2841	$C_{19}H_{39}O_4^+$	331.2843	−0.6
Compound **64** (putatively new)	9.0	358.3105	$C_{24}H_{40}NO^+$	358.3104	+0.3
Compound **65** (putatively new)	9.1	324.3260	$C_{21}H_{42}NO^+$	324.3261	−0.3
Compound **66** (putatively new)	9.2	359.3153	$C_{21}H_{43}O_4^+$	359.3156	−0.8
Compound **67** (pheophorbide-like)	9.4	623.2865	$C_{36}H_{39}N_4O_6^+$	623.2864	+0.1

Compound **68** (putatively new)	9.8	326.3415	$C_{21}H_{44}NO^+$	326.3417	−0.6
Compound **69** (putatively new)	9.9	371.3148	$C_{22}H_{43}O_4^+$	371.3156	−2.1
Compound **70** (putatively new)	10.1	321.3145	$C_{22}H_{41}O^+$	321.3152	−2.2
Lamellodyidea herbacea **(0194-24c)**					
Compound **71** (putatively new)	11.7	510.7661	$C_{13}H_6O_7Br_3^-$	510.7658	+0.6
Compound **72** (putatively new)	13.4	372.7712	$C_7H_4O_3Br_3^-$	372.7705	+1.9
Compound **73** (2,3-dibromo-5-hydroxyphenol)	13.8	264.8501	$C_6H_3O_2Br_2^-$	264.8494	+2.6
Compound **74** (putatively new)	14.0	530.7073	$C_{12}H_7O_4Br_4^-$	532.7072	+0.1
Compound **75** (putatively new)	15.4	342.7607	$C_6H_2O_2Br_3^-$	342.7599	+2.1
Compound **76** (putatively new)	16.8	420.6710	$C_6HO_2Br_4^-$	420.6705	+1.3
Compound **77** (putatively new)	16.8	371.7631	$C_7H_3O_3Br_3^-$	371.7627	+1.2
Compound **78** (putatively new)	17.3	748.6282	$C_{18}H_{10}O_8Br_5^-$	748.6287	−0.6
Compound **79** (putatively new)	18.4	606.6028	$C_{12}H_4O_4Br_5^-$	606.6021	+1.1
Compound **80** (putatively new)	19.6	556.6868	$C_{13}H_5O_5Br_4^-$	556.6865	+0.5
Compound **81** 2,5-dibromo-6-(3',5'-dibromo-2′-hydroxyphenoxy)phenol or 2,4,5-tribromo-6-(5'-bromo-2′-hydroxyphenoxy)phenol	20.9	512.6971	$C_{12}H_5O_3Br_4^-$	512.6967	+0.8
Compound **82** (putatively new)	21.3	620.6182	$C_{13}H_6O_4Br_5^-$	620.6177	+0.7
Compound **83** (2,3,4-tribromo-6-(3',5'-dibromo-2′-hydroxyphenoxy)phenol or 2,4,5-tribromo-6-(3',5'-dibromo-2′-hydroxyphenoxy)phenol or 2,3,5-tribromo-6-(3',5'-dibromo-2′-hydroxyphenoxy)phenol or 3,4,5-tribromo-6-(3',5'-dibromo-2′-hydroxyphenoxy)phenol)	21.6	590.6076	$C_{12}H_4O_3Br_5^-$	590.6072	+0.7
Compound **84** (2,3,4,5-tetrabromo-6-(3',5'-dibromo-2′-hydroxyphenoxy)phenol)	22.6	668.5183	$C_{12}H_3O_3Br_6^-$	668.5177	+0.9
Compound **85** (3,5,6-tribromo-2-(2′-bromophenoxy)phenol or 3,4,5-tribromo-2-(2′-bromophenoxy)phenol or 3,4,6-tribromo-2-(2′-bromophenoxy)phenol or 5,6-dibromo-2-(2′,4'-dibromophenoxy)phenol or 4,6-dibromo-2-(2′,4'-dibromo phenoxy)phenol or 3,6-dibromo-2-(2′,4'-dibromophenoxy)phenol or 3,4-dibromo-2-(2′,4'-dibromophenoxy)phenol or 3,5-dibromo-2-(2′,4'-dibromo phenoxy)phenol)	23.2	496.7023	$C_{12}H_5O_2Br_4^-$	496.7017	+1.2
Compound **86** (2,3,4,5-tetrabromo-6-(3',5'-dibromo-2′-hydroxyphenoxy)phenol)	23.2	668.5181	$C_{12}H_3O_3Br_6^-$	668.5177	+0.5
Compound **87** (2,3,5-tribromo-6-(3',5'-dibromo-2′-phenoxy)anisole or 2,3,5-tribromo-6-(3',5'-dibromo-2′-methoxyphenoxy)phenol or 3,4,5-tribromo-6-(3',5'-dibromo-2′-methoxyphenoxy)phenol or 3,4,5-tribromo-6-(3',5'-dibromo-2′-phenoxy)anisole	23.5	604.6234	$C_{13}H_6O_3Br_5^-$	604.6228	+0.9
